# Titanium Self‐Intercalation in Titanium Diselenide Devices: Insights from In Situ Transmission Electron Microscopy

**DOI:** 10.1002/adma.202418557

**Published:** 2025-03-04

**Authors:** Hsin‐Ya Sung, Che‐Hung Wang, Mu‐Pai Lee, Yu‐Chuan Lin, Yen‐Fu Lin, Chun‐Wei Huang, Wen‐Wei Wu

**Affiliations:** ^1^ Department of Materials Science and Engineering National Yang Ming Chiao Tung University Hsinchu 30010 Taiwan; ^2^ Department of Physics National Chung Hsing University Taichung 40227 Taiwan; ^3^ Department of Material Science and Engineering Institutes of Nanoscience i‐Center for Advanced Science and Technology (i‐CAST) National Chung Hsing University Taichung 40227 Taiwan; ^4^ Department of Materials Science and Engineering Feng Chia University Taichung 407102 Taiwan; ^5^ Center for the Intelligent Semiconductor Nano‐system Technology Research National Yang Ming Chiao Tung University Hsinchu 30010 Taiwan

**Keywords:** 2D cross‐sectional, high‐resolution TEM, in‐situ biasing, self‐intercalation, STEM‐EELS, structural transformation

## Abstract

Metallic transition metal dichalcogenides (MTMDCs) are of significant attention for various electronic applications due to their anisotropic conductivity, high electron mobility, superconductivity, and charge‐density‐waves (CDW). Understanding the correlations between electronic properties and structural transformations is crucial. In this study, a bias‐induced structural transformation in vertical CDW‐based 1T‐TiSe_2_ devices, transitioning from a 1T metallic phase to a distorted transition 1T_d_ phase and subsequently to an orthorhombic Ti_9_Se_2_ conducting phase, is reported. Using ex‐situ and in‐situ biasing transmission electron microscopy, dynamic structural changes, while electron energy loss spectroscopy analysis revealed valence state modifications in Ti and Se within the Ti‐rich layer after biasing, are observed. In addition, the effect of varying 1T‐TiSe_2_ thickness on the maximum current value is investigated. These observations reveal that increased thickness requires higher voltage to induce phase transitions. These insights contribute to understanding the structural and electronic dynamics of 1T‐TiSe_2_, highlighting its potential as a promising material for future CDW‐based device applications.

## Graphical abstract

This study investigates bias‐induced structural transformations in 1T‐TiSe_2_ devices, focusing on the transition from the 1T metallic phase to the distorted 1T_d_ phase and ultimately to an orthorhombic Ti_9_Se_2_ conducting phase. Using ex‐situ and in‐situ TEM, dynamic structural changes and insights into the effect of thickness on phase transitions, providing valuable information for CDW‐based device applications, are revealed.

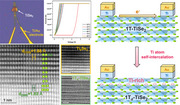

## 1 Introduction

2D MTMDCs, such as TaS_2_,^[^ ^1^, ^2^ ^]^ TiSe_2_,^[^ ^3^ ^]^ NbSe_2_,^[^ ^4^, ^5^, ^6^ ^]^ and VS_2_,^[^ ^7^, ^8^ ^]^ exhibit significant physical properties^[^ ^9^ ^]^ in their bulk states, including magnetism,^[^ ^10^ ^]^ unconventional superconductivity,^[^ ^11^ ^]^ and CDW.^[^ ^12^ ^]^ These CDW phases are characterized by periodic modulation of electron density, temperature dependence, lattice distortion, phonon softening, collective excitations, anisotropic behavior, and nonlinear electrical transport. Additionally, they find applications in hydrogen evolution reactions,^[^ ^13^ ^]^ lithium‐ion capacitors,^[^ ^14^ ^]^ supercapacitors,^[^ ^15^ ^]^ and electronic devices.^[^ ^16^, ^17^ ^]^ The majority of studies on CDW phase materials focus on temperature‐induced plane‐view structural evaluation and the formation of the Star of David (SoD) structure.^[^ ^18^ ^]^ At temperatures above 550 K, the material exhibits a metallic phase, transitioning to an incommensurate CDW (ICCDW) phase below 550 K, a nearly commensurate CDW (NCCDW) phase below 350 K, and finally a commensurate CDW (CCDW) phase below 180 K.^[^ ^19^ ^]^

TiSe_2_, a typical CDW‐based transition metal dichalcogenide (TMD) material,^[^ ^20^ ^]^ belongs to the Group IV layered TMD (LTMD).^[^ ^21^, ^22^ ^]^ It is notable for its strong electron‐electron correlations and exhibits a range of fascinating quantum phases,^[^ ^23^ ^]^ positioning it as a promising electronic substrate material beyond graphene. In addition, the electronic ground state of TiSe_2_ can be readily tuned through chemical intercalation,^[^ ^24^, ^25^ ^]^ pressure,^[^ ^26^ ^]^ and electrical gating.^[^ ^27^ ^]^ LTMD intercalation compounds have garnered considerable research interest,^[^ ^28^ ^]^ particularly in low‐dimensional structures where metal‐metal bonding is critical for synthesizing materials with metallic lattice modulations or CDW.^[^ ^29^ ^]^

In recent decades, advanced in‐situ transmission electron microscopy (TEM) and scanning TEM (STEM) techniques have been utilized to observe samples under external stimuli like heat^[^ ^30^, ^31^ ^]^ and bias.^[^ ^32^, ^33^ ^]^ These methods have significantly enhanced our understanding of electron transport in resistive random‐access memories (RRAM),^[^ ^34^, ^35^ ^]^ nano‐twinned copper electromigration,^[^ ^36^ ^]^ lithium‐ion reactions in batteries,^[^ ^37^ ^]^ and the properties of 2D materials.^[^ ^38^, ^39^, ^40^ ^]^ However, the preparation of cross‐sectional specimens for in‐situ biasing and heating observations in 2D materials remains challenging, often limiting studies to plane‐view observations.

To address this limitation, we conducted ex‐situ temperature‐variable electrical measurements on the 1T‐TiSe_2_ device and performed cross‐sectional analyses both before and after these measurements. Additionally, by utilizing in‐situ atomic resolution TEM, we directly observed the bias‐induced cross‐sectional structural evolution and provided a detailed microstructural analysis. This atomic‐scale insight, obtained through high‐resolution imaging, enabled a thorough examination of structural changes driven by electrical bias. By integrating high‐resolution TEM (HRTEM) and atomic‐resolution annular dark‐field STEM (ADF‐STEM) imaging with electron energy loss spectroscopy (EELS) analysis, we propose a comprehensive mechanism for Ti self‐intercalation and its associated structural transformations. This study not only advances the understanding of CDW‐based Group IV MTMDCs but also establishes a foundation for their application in electronic devices, enhancing the feasibility of cross‐sectional observations in 2D materials.

## 2 Results and Discussion

### 2.1 CDW‐Based Multilayer 1T‐TiSe_2_ Device Investigation

**Figure 1:**
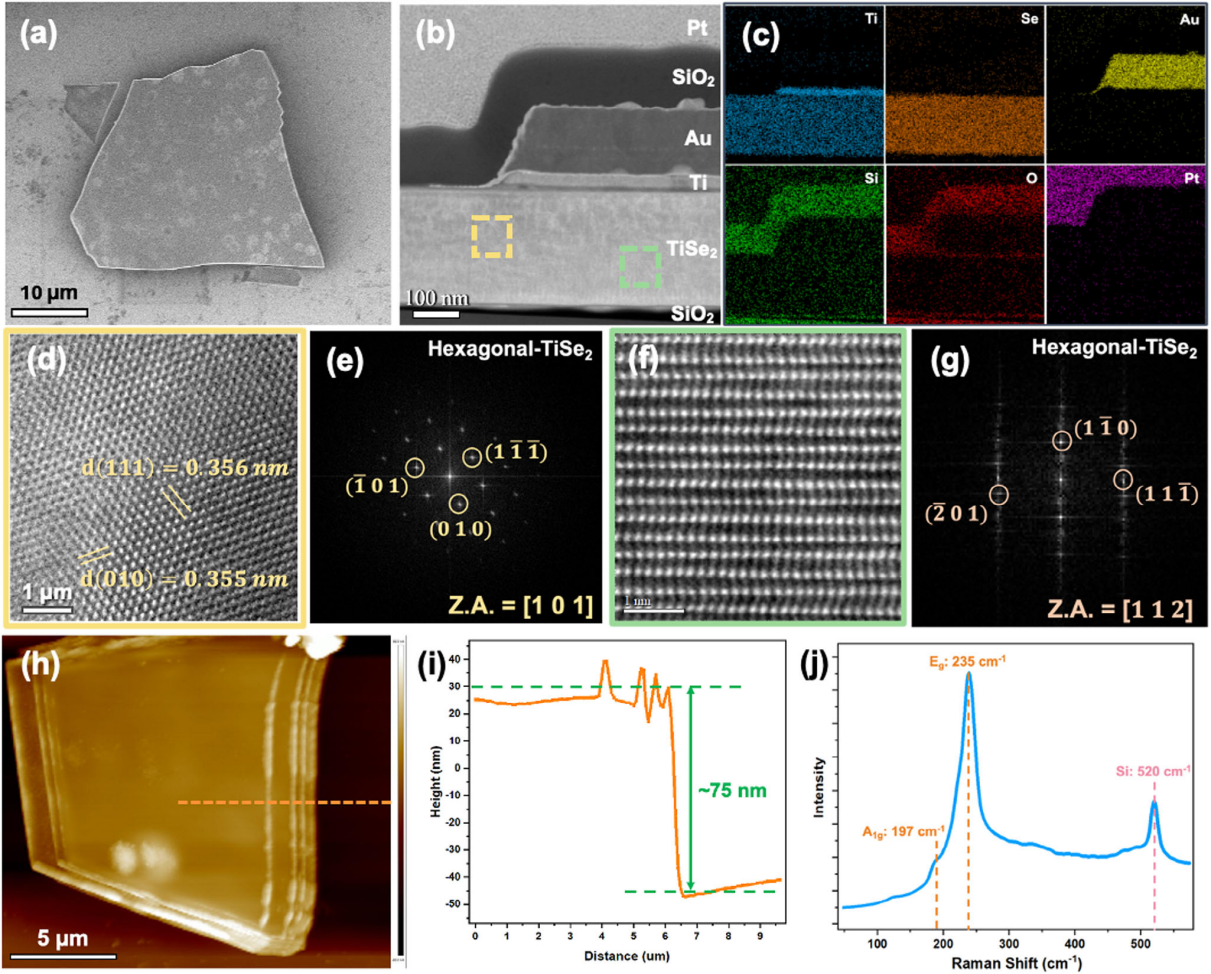
Vertical CDW‐based 1T‐TiSe_2_ device characterization. a) SEM image of pristine 1T‐TiSe_2_ flakes. b) HAADF‐STEM image of a vertical CDW‐based 1T‐TiSe_2_ device showing Ti/Au electrodes, SiO_2_ isolation layer, and Pt protection layer. c) EDS mapping of the vertical CDW‐based 1T‐TiSe_2_ device. d) HRTEM image from the yellow‐boxed region in (b). e) FFT‐DP obtained from (d) along the [101] zone axis. f) High‐magnification HAADF‐STEM image from the green‐boxed region in (b). g) FFT‐DP obtained from (f) along the [112] zone axis. h) AFM topography of exfoliated 1T‐TiSe_2_ flakes. i) Height of the 1T‐TiSe_2_ flake extracted from the AFM topography, indicated by the orange line in (h). j) Raman spectra of a pristine 1T‐TiSe_2_ flake.

High‐quality multilayer TiSe_2_ was transferred onto a Si substrate with a 300‐nm‐thick SiO_2_ layer using the mechanical exfoliation method. **Figure** **1**a shows a scanning electron microscope (SEM) image of a pristine TiSe_2_ flake. The electrodes were initially defined using electron beam lithography (e‐beam lithography) and subsequently deposited via an electron gun (e‐gun) evaporation system. The specimen for TEM observation was subsequently prepared using a focused ion beam (FIB) system, as shown in Figures S1 and S2a–d (Supporting Information). Figure 1b,c displays cross‐sectional high‐angle ADF‐STEM (HAADF‐STEM) images and energy dispersive X‐ray spectroscopy (EDS) mapping of a vertical CDW‐based 1T‐TiSe_2_ device, illustrating the Ti (20 nm)/Au (90 nm) electrodes, the SiO_2_ protection layer, and an ≈250 nm thick TiSe_2_ layer, additional EDS points are shown in Figure S3 (Supporting Information). Notably, an ≈20 nm thick dark line is observed between Ti and TiSe_2_, resulting from Ti's tendency to spontaneously form a stable TiO_2_ layer upon air exposure. This oxide layer protects the electrode from further oxidation and prevents direct interaction between Ti and TiSe_2_, enhancing interface stability. In Figure 1(d), HRTEM image demonstrates that the 1T‐TiSe_2_ is a single crystal with its structure identified through fast Fourier transform diffraction pattern (FFT‐DP) analysis in Figure 1e, showing a hexagonal structure corresponding to the space group $P\bar{3}m1$. Figure 1f presents an ADF‐STEM image for detailed structural analysis, revealing that in the 1T phase, the Ti atom is octahedrally coordinated with six Se atoms with the Se‐Ti‐Se in an ABC stacking fashion. The structure was confirmed by FFT‐DP, as shown in Figure 1g, indicating that 1T‐TiSe_2_ was aligned along the [112] zone axis. Figure 1h,i shows an Atomic Force Microscopy (AFM) micrograph and its height profile, respectively, demonstrating that the 1T‐TiSe_2_ flake has an average height of ≈75 nm. The mechanical exfoliation method allowed control over the thickness of the 1T‐TiSe_2_ flakes are shown in Figure S2e–h (Supporting Information), leading to variability in the thickness of each flake. Raman spectroscopy was performed in a backscattering configuration under λ = 532 nm laser excitation. As shown in Figure 1j, the Raman spectra of the TiSe_2_ flake features peaks at 197 cm^−1^ and 235 cm^−1^, which are attributed to the out‐of‐plane (A_1__g_) and in‐plane (E_g_) Raman modes, respectively. These observed peaks are consistent with previously reported theoretical and experimental values.^[^ ^41^, ^42^, ^43^ ^]^

### 2.2 Ex Situ Structural Evolution in Bias‐Induced in Multilayer 1T‐TiSe_2_ Devices

**Figure 2:**
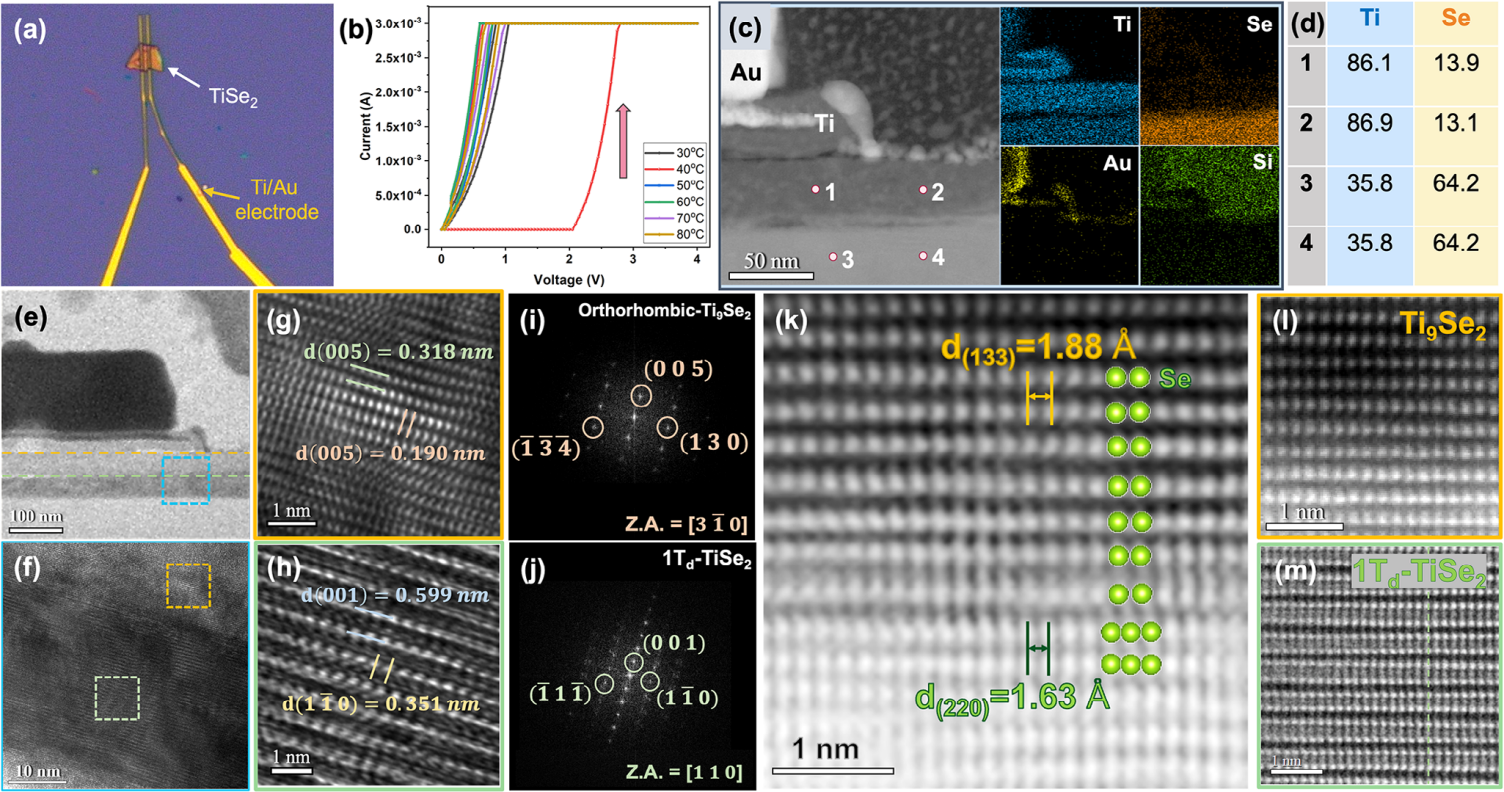
Ex‐situ characterization of the cross‐sectional 1T‐TiSe_2_ device. a) Optical image of exfoliated 1T‐TiSe_2_ after deposition on Ti/Au (20 nm/90 nm) electrodes on a Si/SiO_2_ substrate. b) *I–V* curves of the electroforming process for CDW‐based 1T‐TiSe_2_ devices across temperatures from 30 to 80 °C. c) HAADF‐STEM image and EDS mapping results of a vertical CDW‐based 1T‐TiSe_2_ device after cycling, showing changes near the Ti/Au electrode. d) EDS point results of the cycled sample in (c). e) Low‐magnification TEM image of the cycled sample. f) High‐magnification TEM image from the blue‐boxed region in (e). g) HRTEM image from the region defined by the yellow box in (f). h) HRTEM image from the yellow‐boxed region in (f). i) Corresponding FFT‐DP obtained from (g), showing orthorhombic‐phase Ti_9_Se_2_ along the [$3\bar{1}0$] zone axis. j) FFT‐DP obtained from (h) showing a distorted structure 1T_d_ along the [110] zone axis. k) High‐magnification HAADF‐STEM image of cycled 1T‐TiSe₂ layer, illustrating the interface region between two distinct atomic arrangements. l) High‐magnification HAADF‐STEM image of the upper region in (k), showing orthorhombic‐phase Ti_9_Se_2_. m) High‐magnification HAADF‐STEM image from the lower region of (k) showing the 1T_d_‐TiSe_2_ structure.

The Ex‐situ electrical measurement device as shown in **Figure** **2**a. The *I–V* curve of the electroforming process for the 1T‐TiSe_2_ device was recorded over a temperature range from 30 to 80 °C in Figure 2b. The voltage was incrementally increased by 0.05 V every second until reaching a maximum of 4 V. A sharp increase in current at 40 °C, indicated by the pink arrow on the curve, suggests the presence of a threshold switching effect, a phenomenon commonly observed in other CDW materials.^[^ ^44^ ^]^ Raman analysis of the device after the ex‐situ measurements showed significant changes in the intensity and positions of the characteristic peaks, which indicate possible structural transitions. This distinction allows for a comparative analysis of the structural and electrical changes between the biased and non‐biased regions are shown in Figure S4 (Supporting Information). To further explore the relationship between electrical properties and atomic structure changes in the 1T‐TiSe_2_ device, cross‐sectional observations were conducted, with detailed low‐to‐high‐magnification images in Figure S5 (Supporting Information). Figure 2c shows that the initial 1T‐TiSe_2_ layer was divided into two layers near the electrode after measurement, with EDS mapping analysis identifying a Ti‐rich area in the upper layer. The quantitative analysis in Figure 2d shows that the Ti ratio in the upper part had increased significantly, while the lower part retained the original Ti to Se stoichiometric ratio of 1:2.

Additional structural information for the observed delamination area, we utilized HRTEM, the corresponding FFT‐DP, along with HAADF‐STEM imaging. Figure 2e shows a low‐magnification TEM image of the cycled sample, with a detailed higher‐resolution view provided in Figure 2f. Figure 2g–i showing HRTEM images and corresponding FFT‐DP, indicate that the upper Ti‐rich region adopts a Ti_9_Se_2_ orthorhombic crystal structure with space group Pbam. In contrast, the lower part transforms into a structure similar to the 1T phase, as shown in Figure 2h–j, which is defined as the distorted transition 1T_d_ structure. Notable deviations in d‐spacing and FFT‐DP results between the 1T_d_ and 1T phases were observed. Subsequently, as shown in Figure 2k, the HAADF‐STEM images revealed distinct arrangements of atomic structures and variations in d‐spacing are shown in Figure S6 (Supporting Information). As illustrated in Figure 2l, the Ti_9_Se_2_ orthorhombic phase in the upper region near the electrode surface promotes conductive pathways, thereby enhancing current flow within the material. Similarly, the lower part of TiSe_2_, as shown in Figure 2m, exhibits a transition from the original metallic 1T phase to the 1T_d_ phase (an intermediate state) between the 1T and metal‐rich phases, involving a shift in the atomic arrangement from the original ABC stacking to ABA stacking, similar to the semiconductor 2H phase. In regions farther from the electrode, incomplete reactions preserved the original atomic structural arrangement are shown in Figure S7 (Supporting Information). Similar intermediate‐state behaviors have also been observed in previous RRAM studies on conductive filament formation.^[^ ^45^ ^]^

### 2.3 In Situ Bias‐Induced Transformation from 1T to a Titanium‐Rich Phase

After investigating the bias‐induced phase transition in ex‐situ 1T‐TiSe_2_, we further conducted in‐situ observations to examine the phase transformation in 1T‐TiSe_2_ under bias. Cross‐sectional lamellae of the 1T‐TiSe_2_ sample were prepared using FIB techniques for these observations. The lamella was then transferred to an in‐situ electrical TEM chip with a glass tip, and Pt wires were deposited using the FIB system to establish electrical connections between the sample and the chip. A specialized in situ TEM holder was used to apply a bias to the sample for further analysis. The detailed setup of in‐situ experiments are shown in Figures S8 and S9a,b (Supporting Information). The in‐situ biasing experiment was performed at 250 k time magnification. The applied voltage was increased by 0.05 V per second until the target voltage was reached. The bias voltage was then increased to 2 V and maintained for 15 min, followed by an increase to 2.5 V for an additional 5 min, the voltage versus time profile is shown in Figure S9c (Supporting Information). During the in‐situ biasing experiment, the *I–V* curve demonstrated a significant increase in current as shown in Figure S9d (Supporting Information), reaching ≈1.5 × 10^−4^ A at 2.5 V, which was consistent with the observation from ex‐situ measurements.

**Figure 3:**
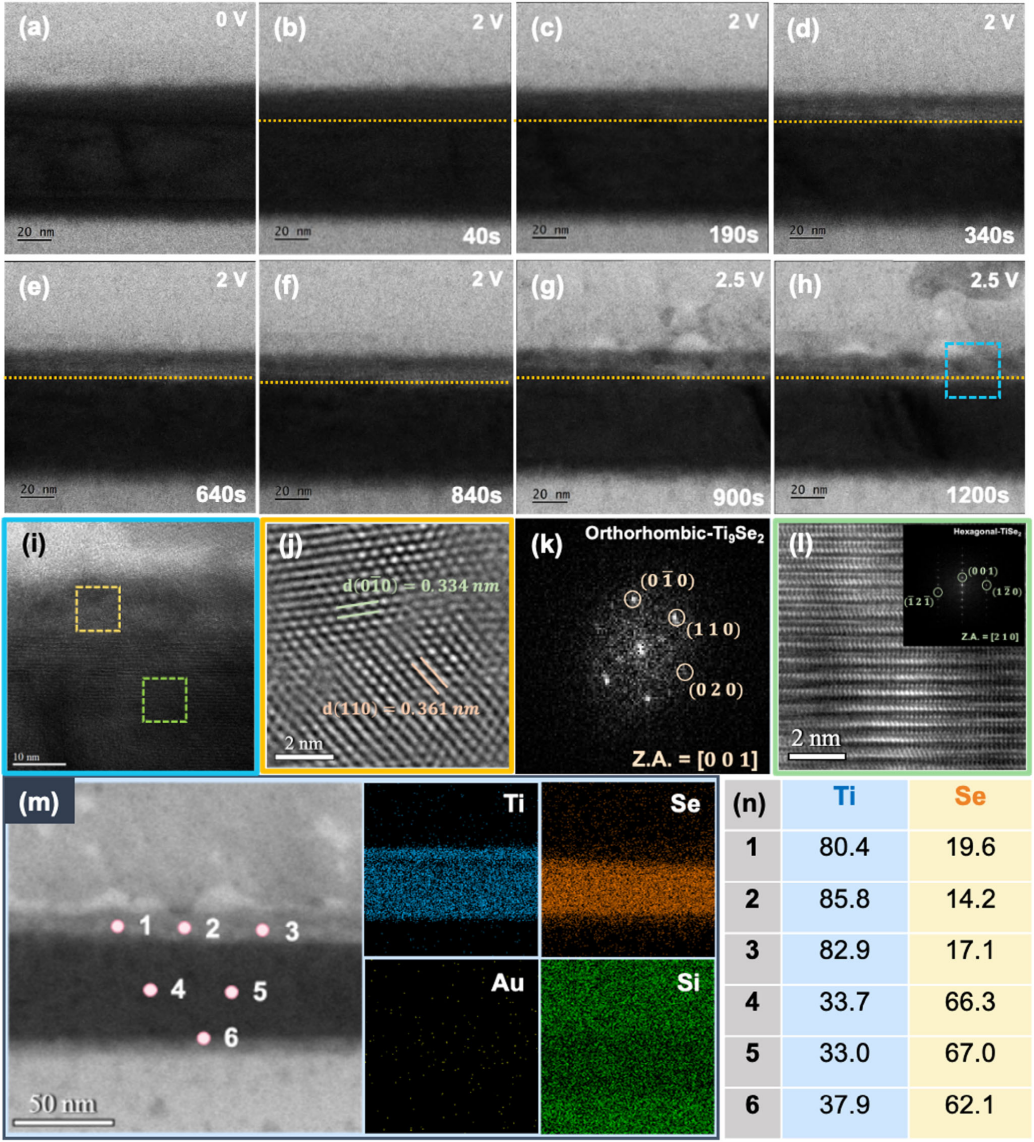
In‐situ biasing investigation of the cross‐sectional 1T‐TiSe_2_ device. a–h) Time‐sequenced TEM images illustrating the bias‐induced phase evolution in the 1T‐TiSe_2_ device under a 2.5 V bias. As the bias increases, the contrast changes in the 1T‐TiSe_2_ layer are indicated by the yellow dashed area. i) High‐magnification TEM image from the blue‐boxed region in (h). j) HRTEM image from the yellow‐boxed region in (i). k) Corresponding FFT‐DP image from j, showing the orthorhombic‐phase Ti_9_Se_2_ along the [001] zone axis. l) HRTEM image of the green‐boxed region in (i), with the inset showing the corresponding FFT‐DP. m) HAADF‐STEM image, and EDS mapping of the biased 1T‐TiSe_2_ device, showing the elemental distributions of Ti, Se, Au, and Si. n) EDS point results of the biased 1T‐TiSe_2_ device in (m).

To demonstrate the structural changes induced by the bias voltage, advanced in‐situ TEM techniques were employed. An initial characterization of the material was performed prior to biasing are shown in Figures S10 and S11 (Supporting Information). Subsequently, we analyzed time‐sequenced TEM images shown in **Figure** **3**a–h (see Movie S1, Supporting Information, for the complete in‐situ process) and tracked the changes over time after biasing. When the voltage was maintained at 2 V, the upper layer of 1T‐TiSe_2_ began to exhibit changes after ≈190 s. As the voltage increased, these transformations became more evident. After the completion of the reaction at 2.5 V, a detailed microstructural analysis revealed the appearance of the Ti_9_Se_2_ structure in the upper layer, as shown in Figure 3i–k. This Ti‐rich phase, which formed during the biasing of the entire device, acts as a conduction path, resulting in an increase in the overall current. In contrast, the lower part of the structure retained the original 1T‐TiSe_2_ structure (Figure 3l). Notably, during the in‐situ biasing process, no intermediate 1T_d_ phase was observed due to the absence of stable thermal effects. Figure 3m,n confirms an increased Ti atomic percentage in the upper layer, indicating the formation of a Ti‐rich layer. The in‐situ biasing confines the current flow to the 1T‐TiSe_2_ lamella, allowing it to pass through the entire layer. This may induce phase transformations in regions not directly observed by the electron beam, as shown in Figure S12 (Supporting Information). Consequently, the phase transformation is primarily driven by the current rather than the electron beam effect. Furthermore, while Joule heating during current flow is inherently non‐uniform, the uniformly observed phase changes in this experiment suggest that Joule heating is not the dominant factor. Additionally, no phase change was observed in the in‐situ heating of 1T‐TiSe_2_, as shown in Figure S13 (Supporting Information), indicating that the phase transition is driven solely by the current. In addition, during in‐situ biasing of the thicker device (200 nm), the reaction initiated at 2.5 V, with a current threshold switching effect observed at 4.5 V. The maximum current in the thicker device was an order of Magnitude lower than that in the thinner device (70 nm). Furthermore, thicker devices required a higher voltage for phase transitions and exhibited longer reaction times are shown in Movie S2 and Figure S14 (Supporting Information).

**Figure 4:**
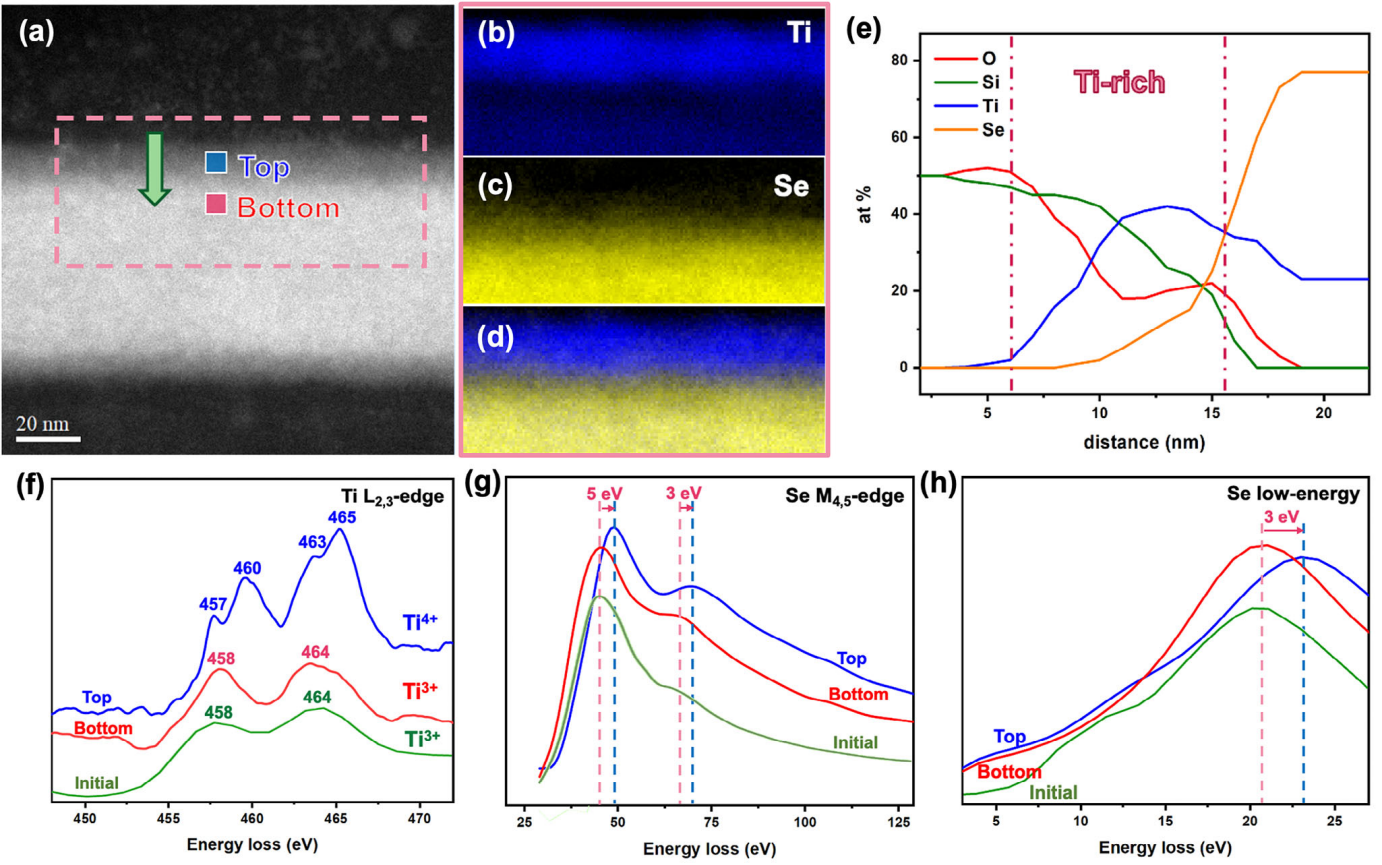
EELS analysis of the 1T‐TiSe_2_ device after in‐situ biasing. a) STEM image of the biased 1T‐TiSe_2_ device, with blue and red frames indicating the area analyzed by EELS. b–d) EELS mapping of the region defined by the pink box in (a), showing the elemental distribution of (b) Ti, (c) Se, and (d) overlapping images. e) EELS line scan along the area indicated by the green arrow in a, showing the elemental distribution along the line. f–h), EELS spectra of (f) Ti‐L_2,3_; (g) Se‐M_4,5_; and (h) Se low‐energy edges for both the initial and biased 1T‐TiSe_2_ devices. The green line represents the initial device, the red line indicates the spectra from the bottom layer of the biased device, and the blue line depicts the spectra from the top layer of the biased device.

To acquire more evidence and achieve a comprehensive understanding of the phase transformation, it was essential to use EELS spectroscopy for a comparative analysis of the pristine and biased devices are shown in Figure S15 (Supporting Information). **Figure** **4** presents the EELS mapping, line scans, and spectra of Ti and Se after biasing. In Figure 4a–d, the EELS mapping analysis focuses on the layered region in the upper half of the biased sample, highlighted by the pink frame. It is evident that the upper half is Ti‐rich, while the lower half is Se‐rich. Additionally, the EELS line scan shown in Figure 4e, indicated by the green arrow, further confirms the presence of regions with a higher concentration of Ti. The valence state of the Ti was determined based on the peak shape of the L_2,3_ edge. As illustrated in Figure 4f, the analysis of the initial layer and its upper and lower halves (marked by green, blue, and red lines, respectively) shows that the lower half maintained the original Ti^3+^ valence state, whereas the upper half transformed to the Ti^4+^ valence state, indicating an increased concentration of Ti atoms in the upper layer. Since metal‐rich conditions typically result in a Ti valence state of 4^+^, this observation confirms the presence of an octahedral bonding configuration, consistent with previous studies.^[^ ^46^, ^47^, ^48^ ^]^ Additionally, as shown in Figure 4g,h and Figure S16 (Supporting Information), compared to the pristine TiSe_2_ region, the M_4,5_ edge and the low‐energy loss spectrum peaks of Se in the metal‐rich region shifted toward higher energy losses. This shift is attributed to the lower Se content, which tends to increase energy losses, aligning with trends observed in earlier research.^[^ ^49^ ^]^

### 2.4 Mechanism of Bias‐Induced Ti Self‐Intercalation

**Figure 5:**
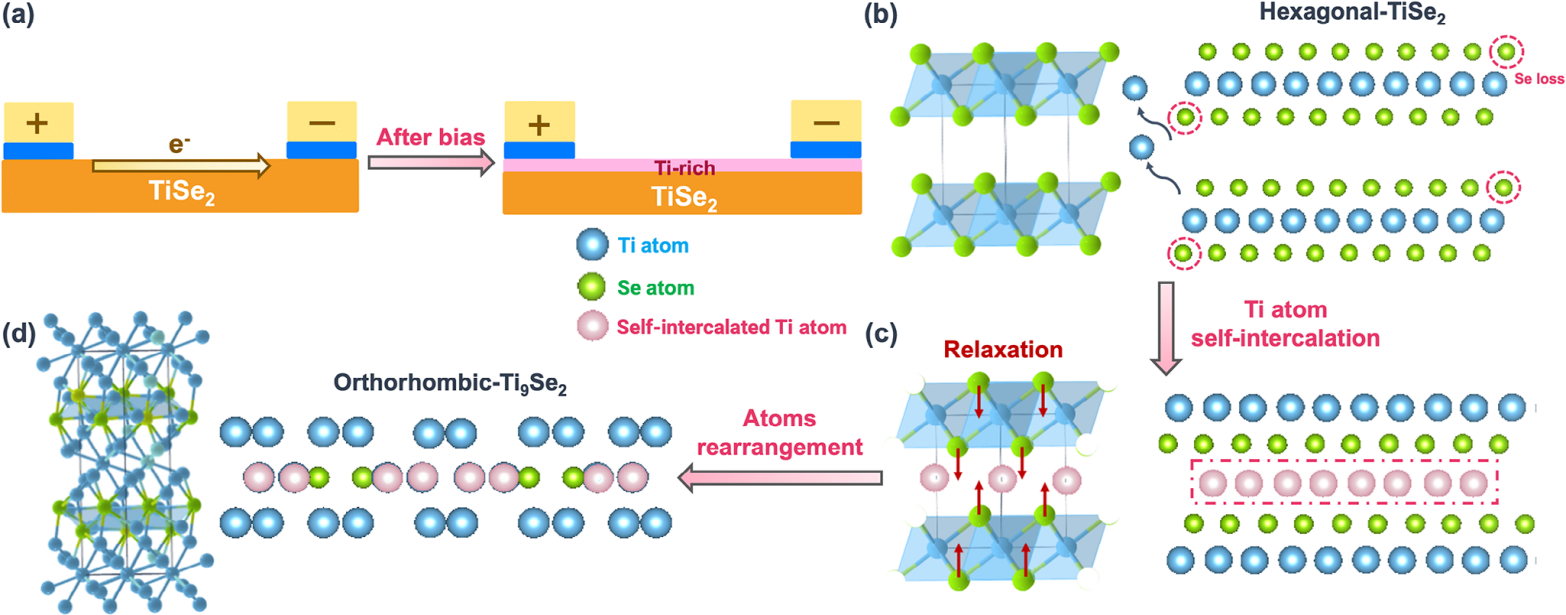
Schematic of the structural transformation process of the 1T‐TiSe_2_ device. a) Schematic of the biasing experiment. b–d) Dynamic structural transformation process of the 1T‐TiSe_2_ reconstruction during biasing. (b) Applied bias induces the migration of Se atoms, resulting in unbonded Ti atoms. (c) Ti atoms self‐intercalate into the distorted 1T‐TiSe_2_ lattice, resulting in lattice reconstruction. (d) Formation of Ti_9_Se_2_ on the device surface after the application of bias voltage.

To investigate the mechanism of the bias‐induced phase transformation in 1T‐TiSe_2_ devices, our study was divided into several steps. The schematic of the biasing shown in **Figure** **5**a illustrates that after biasing, a Ti‐rich phase forms on the surface of the device. As shown in Figure 5b, the initial state of the device consists of multilayered TiSe_2_ with a hexagonal crystal structure. When a voltage bias is applied across the device, the increasing voltage causes the electric fields and currents to interact with the lattice atoms. The bond between Ti and Se in TiSe_2_ is relatively weaker compared to Ti─Ti or Se─Se bonds, allowing Se atoms to move more easily under an electric field. The current can expel selenium atoms,^[^ ^50^, ^51^ ^]^ promoting their migration and causing them to move away from regions of high current density after bond breaking, ultimately leading to selenium loss. Under the influence of a bias voltage, Se atoms gradually migrate toward the electrode and accelerate volatilization due to the Joule heating effect.^[^ ^52^, ^53^ ^]^ Additionally, the formation energy of Se vacancies (0.01 eV) is significantly lower than that of Ti vacancies (2.07 eV),^[^ ^54^ ^]^ making Se atoms more prone to detaching from the crystal lattice and leaving unbound Ti atoms behind.

Furthermore, in Figure S17 (Supporting Information), 4D‐STEM observations before and after applying a 2.5 V bias voltage reveal minimal changes in strain along the Exx direction but significant changes in the Eyy strain. In the original sample, TiSe_2_ exhibits a compact strain distribution, while the bias increases the strain in the upper Ti_9_Se_2_ layer. This suggests that selenium atoms may migrate or volatilize due to electromigration, creating defects that trigger lattice reconstruction and an increase in Eyy strain. Meanwhile, the lower TiSe_2_ layer shows a more relaxed strain distribution.

Once the voltage reaches a critical value (typically ≈2.5 V), the energy provided by the electric field and current is sufficient to overcome the activation energy required for Ti atom mobility. Consequently, Ti atoms begin migrating through the lattice. This migration is facilitated by the creation of Se vacancies or interstitial sites within the lattice, allowing Ti atoms to move into the van der Waals interlayer of the bilayer 1T‐TiSe_2_ and occupy octahedral site positions.^[^ ^55^ ^]^ As shown in Figure 5c, this process, known as Ti self‐intercalation, significantly alters the properties of the material and is thermodynamically favorable.^[^ ^55^ ^]^ With the migration of additional Ti atoms, a phase transformation occurs within the material, leading to substantial changes in its electronic properties. In the final state, the device reaches a new equilibrium, where the concentration of inserted Ti atoms is higher occupying multiple octahedral sites and forming metal‐metal‐bonded octahedral M_6_ cores.^[^ ^56^, ^57^ ^]^ This resulted in the formation of Ti_9_Se_2_ with an orthorhombic crystal structure, as shown in Figure 5d. This new structure features a direct energy gap, enhancing electrical conductivity and significantly increasing current flow through the device, thereby imparting metallic electrical properties to the device. Based on both thermodynamic and kinetic considerations, we offer an in‐depth discussion of the bias‐induced Ti self‐intercalation behavior in CDW‐based 1T‐TiSe_2_ devices, as well as the associated phase transformations.

## 3 Conclusion

In summary, we presented novel cross‐sectional observations of the structural transformation of 1T‐TiSe_2_ devices under biasing. The ex‐situ measurements, driven by both heat and bias, revealed the appearance of a thermodynamically unstable 1T_d_ phase within the structure. As the voltage applied to the 1T‐TiSe_2_ device increased under bias conditions, the threshold‐switching effect induced a substantial increase in current. This change in electrical properties is strongly related to the crystal structure, highlighting the intricate relationship between structural and electronic behaviors. Specifically, the weaker Ti─Se bond in TiSe_2_, combined with the low vacancy formation energy of Se, enables Se atoms to vacate their original crystal sites, volatilize under Joule heating, and leave behind unbonded Ti atoms. These Ti atoms subsequently self‐intercalate into the van der Waals interlayer octahedral sites of 1T‐TiSe_2_, forming a layer of orthorhombic Ti_9_Se_2_ composed of multiple octahedral sites. This Ti self‐intercalation process is thermodynamically favorable, leading to the creation of a Ti‐rich layer that significantly enhances electron conduction within the device. Consequently, the device exhibits faster charging and increased current flow. Overall, this comprehensive study of bias‐induced material transformations provides valuable insights for the future design and reliable application of CDW‐based devices.

## 4 Experimental Section

### Preparation of 1T‐TiSe_2_ Flakes

Pristine 1T‐TiSe_2_ flakes were prepared by mechanical exfoliation. Bulk TiSe_2_ adhered to blue tape, which was folded 5–10 times to produce flakes of different thicknesses. The tape was then placed onto a Si substrate with a 300‐nm‐thick SiO_2_ layer and pressurized for ≈10 s before removal. The 1T‐TiSe_2_ flakes were then observed under an optical microscope.

### Fabrication of 1T‐TiSe_2_ Devices

MAA (Copolymer) and polymethyl methacrylate (PMMA) were spin‐coated onto the chip as positive‐tone photoresist (PR), followed by baking at 150 and 180 °C for 1.5 min using electron beam lithography (ELS‐7500EX). Ti (20 nm) and Au (90 nm) were deposited as electrodes using an e‐gun evaporation system. Subsequently, PR was removed by lift‐off using acetone for 24 h.

### Electrical Properties Measurement

The ex‐situ electrical characteristics of the 1T‐TiSe_2_ devices were measured using a semiconductor parameter analyzer (Agilent 4145 B and B1500 systems) over a temperature range of 30–80 °C. During measurements, a voltage source was applied to the Ti/Au electrodes to operate the devices. The *I–V* curves were obtained using direct voltage sweeps with a voltage step of 0.05 V s^−1^.

### Microstructural Characterization of 1T‐TiSe_2_

The TiSe_2_ flakes were characterized by optical microscopy (OM), SEM, AFM, and Raman spectroscopy to study their morphology, determine sample thickness, and analyze the characteristics of 1T‐TiSe_2_. Raman spectroscopy (Horiba Jobin Yvon Labram HR 800) was conducted with an excitation wavelength of 532 nm. A dual‐beam FIB microscope system (TESCAN Lyra3) was employed to fabricate the cross‐sectional ex‐situ H‐shaped TEM samples. To protect the device from damage by the Ga ion beam during subsequent procedures, a Pt protective layer (10 µm × 1.5 µm × 1.5 µm) was deposited onto the device surface using the FIB system. The prepared TEM samples were carefully transferred to a Cu grid using a glass tip. The structural evolution of the 1T‐TiSe_2_ device was directly observed using field‐emission TEM (JEOL‐F200) at an accelerating voltage of 200 kV. The elemental distribution was analyzed using EDS (Oxford EDS 100 TLE). Additionally, EELS spectra and STEM images were obtained using a Cs‐corrected TEM (JEOL JEM‐ARM200FTH) at an accelerating voltage of 200 kV.

### TEM/STEM with a Specialized TEM Holder and Image Processing

A dual‐beam FIB system (TESCAN Lyra3) was used to prepare cross‐sectional in‐situ TEM samples with a width of 12 µm. To prevent Ga ion damage, a Pt protection layer was deposited on the TiSe_2_ surface (Figure S8b, Supporting Information). Trenches were milled on both sides of the lamella (Figure S8c, Supporting Information), followed by a low‐kV cleaning step (Figure S8d, Supporting Information) to minimize Ga ion effects. The sample was then transferred onto the in‐situ TEM chip using a glass tip. In‐situ experiments were conducted with a TEM holder (Protochips Aduro 300) and observed using TEM (JEOL JEM‐F200) at 200 kV. For in‐situ biasing, a voltage of 0.05 V s^−1^ was applied, while for in‐situ heating, the temperature was increased at 1 °C s^−1^. The setup included a power supply (2616A System Sourcemeter) and a software controller (Fusion 350 V1.0.0). ADF‐STEM images were acquired using Cs‐corrected TEM (JEOL JEM‐ARM200FTH) at 200 kV, and atomic models were generated with VESTA software.

### 4D‐STEM and Image Processing

Atomic‐resolution strain mapping was performed using a Gatan Stela direct electron detector and the STEMx system, which was integrated into an FEI Titan Chemi‐STEM operating at 200 kV. The system was set with a circular C2 aperture of 10 µm, a convergence angle of 13.8 mrad, a microprobe lens configuration, a spot size of 6, and a camera length of 165 mm. The acquired data were binned to a resolution of 256 × 256 pixels before being subjected to computational analysis. Strain mapping data were processed using the open‐source Gatan Digital Micrograph Software.

## Conflict of Interest

The authors declare no conflict of interest.

## Supporting information



Supporting Information

Supplemental Movie 1

Supplemental Movie 2

## Data Availability

The data that support the findings of this study are available from the corresponding author upon reasonable request.
