# Supplementary material for: Titanium Self‐Intercalation in Titanium Diselenide Devices: Insights from In Situ Transmission Electron Microscopy
Source: Adv Mater. 2025 Mar 4;37(17):2418557. doi: 10.1002/adma.202418557 (PMC12038540; doi:10.1002/adma.202418557)
Supplement: Supplementary file 1 — Supporting Information [file ADMA-37-2418557-s001.docx]

Supporting Information

**Titanium Self-Intercalation in Titanium Diselenide Devices: Insights from *In Situ* Transmission Electron Microscopy**

*Hsin-Ya Sung*, *Che-Hung Wang*, *Mu-Pai Lee*, *Yu-Chuan Lin,* *Yen-Fu Lin*, *Chun-Wei Huang*, *Wen-Wei Wu* ***

**List of contents**

Figure S1｜**Experimental setup for *in-situ* and *ex-situ* samples.**

Figure S2｜**Initial identification of pristine 1T-TiSe_2_.**

Figure S3｜**Characterizations of the pristine cross-section 1T-TiSe_2_ device.**

Figure S4｜**Raman spectra of the 1T-TiSe_2_ device after *ex-situ* biasing.**

Figure S5｜**Detailed TEM images of the cross-sectional 1T-TiSe_2_ device after *ex-situ* electrical measurements.**

Figure S6｜**ADF-STEM images of the e*x-situ* biased1T-TiSe_2_ device.**

Figure S7｜***Ex-situ* observations of the cross-sectional 1T-TiSe_2_ device away from the electrode.**

Figure S8｜***Ex-situ* and *in-situ* TEM lamella preparation using the FIB system.**

Figure S9｜**Pre-biasing experimental setup before biasing and electrical performance of the *in-situ* sample.**

Figure S10｜**Initial state of the *in-situ* cross-sectional 1T-TiSe_2_ device.**

Figure S11｜ **Schematic illustration of the *in-situ* cross-sectional 1T-TiSe_2_ device.**

Figure S12｜**Elemental quantitative Analysis of *in-situ* cross-sectional 1T-TiSe_2_ device in the non-electron beam irradiated area after biasing.**

Figure S13｜***In-situ* heating observation of the 1T-TiSe_2_ device.**

Figure S14｜***In-situ* biasing observation of the 200 nm 1T-TiSe_2_ device.**

Figure S15｜ **EELS characterization of the pristine state 1T-TiSe_2_ device.**

Figure S16｜ **EELS analysis of the *in-situ* biased 1T-TiSe_2_ device.**

Figure S17｜**Comparison of strain in pristine and biased 1T-TiSe_2_ devices using 4D-STEM.**

Section S1: Sample preparation for *in-situ* and *ex-situ* lamellas

Bulk TiSe_2_ was transferred onto a silicon substrate with a 300-nm-thick SiO_2_ layer using the mechanical exfoliation method, featuring Ti/Au electrodes at the edge of the substrate, as shown in **Figure S1(a)-(b)**. These electrodes were then defined on the TiSe_2_ flakes using electron beam lithography, followed by the deposition of Ti (20 nm)/Au (90 nm) electrodes *via* an e-gun evaporation system. A100-nm SiO_2_ isolation layer was deposited prior to FIB process to protect the TiSe_2_ from the FIB-induced damage. The main differences between the *in-situ* and *ex-situ* samples are the lamella length and electrode spacing (see **Figure S1(c)-(d)**). For the *in-situ* samples, the lamella length is approximately 12 µm, with an electrode spacing of about 5 µm. This design minimizes the risk of platinum sputtering during FIB electrode deposition, preventing interference with subsequent TEM observations. In contrast, for the *ex-situ* samples, the lamella length is approximately 10 µm. This ensures the lamella lies flat on the copper grid surface, facilitating observation, while the electrode spacing is around 3 µm. **Figure S1(e)-(f)** illustrate the cross-sectional schematics of the *in-situ* and *ex-situ* lamellae, respectively.

Section S2: Fundamental identification of pristine 1T-TiSe_2_

**Figure S2(a)-(b)** present SEM images of TiSe_2_ flakes on the silicon substrate. Using the mechanical exfoliation method, samples with various morphologies and controllable thicknesses were obtained. A low-magnification TEM image of an *ex-situ* lamella reveals the complete cross-sectional TiSe_2_ layer, as shown in **Figure S2(c)-(d)**. AFM micrographs and height profiles of TiSe_2_ flakes with different thicknesses are presented in **Figure S2(e)-(h)**. Further qualitative analysis in **Figure S3** of the original 1T-TiSe₂ device demonstrates the uniform distribution of Ti and Se, maintaining a 1:2 ratio.

Section S3: *Ex-situ* analysis of the phase transition in the 1T-TiSe₂ device

**Figure S4(a)** shows the *ex-situ* biased 1T-TiSe_2_ device with patterned Ti/Au electrodes, while **Figure S4(b)** presents the Raman mapping of the biased device from the region defined by the green box in **Figure S4(a)**. The Raman intensity ratio across the regions revealed distinct patterns, indicating potential reactions within the device. Spectral analysis further demonstrates that (**Figure S4(c)**), in its pristine state (red line), 1T-TiSe_2_ exhibits intrinsic Raman peaks at 199 cm⁻¹ and 235 cm⁻¹. After electrical biasing (blue line), there was a pronounced increase in the intensity of the Raman peak at 235 cm⁻¹, along with the emergence of a new peak at 146 cm⁻¹, suggesting potential structural transitions and CDW modulation. These structural transition signals were confined to the region between the electrodes (region a) where the electrical bias was applied, whereas the regions outside the electrodes (region b) remained unaffected by the current flow, indicating bias-induced Ti intercalation and lattice distortion consistent with phase transformation in the 1T structure under electrical stress.

To clearly illustrate the device for *ex-situ* TEM observation, Figure S5 presents detailed TEM images of the cross-sectional 1T-TiSe_2_ device after *ex-situ* electrical measurements. The low-magnification TEM images in **Figure S5(a)-(b)** depict the overall device structure and electrodes, respectively. A high-magnification TEM image in **Figure S5(c)**, taken from the orange-boxed region in **Figure S5(b)**, highlights the delamination phenomenon of 1T-TiSe_2_ near the electrode after electrical measurements. Additionally, the high-magnification STEM image in **Figure S5(d)**, corresponds to the EDS focus area in **Figure 2(c)**, providing further elemental distribution analysis.

Subsequent analysis of the HAADF-STEM images uncovered distinct atomic arrangements within the layered regions, as shown in **Figure S6(a)-(b)**. Corresponding FFT-DP analysis (**Figure S6(c)**) identifying an orthorhombic Ti_9_Se_2_ phase along the [$0\bar{1}1$] zone axis in the upper layer. In contrast, **Figure S6(d)** reveals a transitional 1T_d_ phase along the [110] zone axis in the lower part of TiSe_2_. However, in the same specimen shown in **Figure 2**, unreacted 1T-TiSe_2_ layers were found in areas far from the electrode (**Figure S7(a)-(b)**), which maintained the original atomic arrangement (**Figure S7(c)-(f)**), suggesting a random distribution of reaction zones across the flakes.

Section S4: *In situ* biasing experiments setup

**Figure S8(a)-(d)** llustrate the differences in the morphologies of the *ex-situ* and *in-situ* TEM lamellae prepared using the FIB system, with widths of 10 μm and 12 μm, respectively. The top view of the *in-situ* sample was modified to a U-shape to facilitate the deposition of Pt wires. The lamella was transferred onto an *in-situ* electrical TEM chip using a glass tip, and a Pt wire was deposited on the FIB system to connect the electrodes between the sample and the chip (**Figure S8(e)-(f)**). The electrical TEM chip was then placed on an *in-situ* TEM holder (Protochips Aduro 300) under bias conditions for electrical measurements performed using DC voltage sweeps (**Figure S9(a)**). **Figure S9(b)** illustrates a schematic of the lamella positioned within the electrical chip observation window.

Section S5: *In situ* biasing analysis of the cross-sectional view of the 1T-TiSe_2_ device

**Figure S9(c)** shows the profile of voltage increase over time, while **Figure S9(d)** presents the *I-V* curve during the *in-situ* process for the 70nm 1T-TiSe_2_ device. The maximum current value was 1.5$\times$10^-4^ A at 2.5 V. Before conducting the *in-situ* analysis, the device in its initial state was examined. **Figure S10(a)** displays a low-magnification view of the device, while **Figure S10(b)-(d)** provide additional confirmation of the 1T-TiSe_2_ layer structure. Furthermore, EDS mapping analysis (**Figure S10(e)-(g)**) demonstrates the uniform distribution of Ti and Se elements. **Figure S10(h)** further verifies that the Ti to Se ratio was approximately 1:2 based on EDS point analysis.

**Figure S11** shows TEM images of the *in-situ* 1T-TiSe_2_ device before and after biasing. **Figure S11(a)** presents a low-magnification TEM image of the initial device, while **Figure S11(b)** provides a high-magnification TEM image of the blue-boxed area in **FigureS11(a)**, highlighting the FIB-created hole in the Pt layer, ensuring that the current is confined to the 1T-TiSe_2_ layer. After biasing, the low-magnification TEM image in **Figure S11(c)** demonstrates the stability of the device, showing that current does not flow through the Pt layer, preventing overheating and melting.

To differentiate between the effects of the current and electron-beam irradiation, **Figure S12** shows the EDS analysis of the area not irradiated by the electron beam, which also exhibited a phase transformation. This clearly demonstrates that the current primarily induced the phase transformation.

Section S6: *In situ* heating of the cross-sectional 1T-TiSe_2_ device

To investigate whether the phase transition is driven by electrical current or thermal effects, we performed *in-situ* heating to evaluate the stability of the 1T-TiSe_2_ device. **Figure S13(a)** shows a schematic of the 1T-TiSe_2_ device placed on the heating chip, while **Figure S13(b)** illustrates the temperature profile during the *in-situ* heating process. The temperature was increased at a rate of 1°C per second until it reached 600°C, where it was held for 10 minutes. After maintaining 600°C for 10 minutes, no significant delamination was observed (**Figure S13(f**)), and EDS quantitative analysis conducted before and after heating (**Figure S13(d)-(e) and S13(g)-(h)**) showed no formation of a Ti-rich phase. These results suggest that the phase transition is mainly driven by current injection, which modifies the electronic state, rather than solely by thermal effects. This further confirms that the phase transition mechanism in CDW materials is primarily influenced by changes in the electronic structure.

Section S7: *In situ* biasing analysis of the thicker 1T-TiSe_2_ device

For the *in-situ* analysis of thicker devices (200 nm), refer to the time-sequenced TEM images in **Figure S14(a)-(f)**, with a voltage increase rate of 0.05 V per second. The detailed experimental parameters are presented in **Figure S14(g)**. Additionally, in **Figure S14(h)**, during the biasing process, changes began to occur at 2.5 V, and when the voltage reached 4.5 V, the current increased dramatically to 1.3$\times$10^-5^ A.

Section S8: EELS results of pristine state 1T-TiSe_2_ device

**Figure S15(a)** shows the ADF-STEM image of the pristine 1T-TiSe_2_ device, with the orange and green frames indicating the EELS analysis areas. EELS mapping from the region defined by the yellow box in **Figure** **S15(a)** confirms the uniform distribution of Ti and Se, as shown in **Figure S15(b)-(d)**. Therefore, the Se low-energy loss spectrum (**Figure S15(e)**) exhibits no significant peak shifts in the 1T-TiSe_2_ layer.

Section S9: EELS results of biased 1T-TiSe_2_ device

**Figure S16(a)** shows the high-magnification EELS spectrum analysis of the biased sample, with the analysis areas indicated by red and blue frames. The Ti L_2,3_ edge spectrum (**Figure S16(b)**) reveals a valence state transition: Ti^3⁺^ in the lower 1T-TiSe_2_ layer and Ti^4⁺^ in the upper metal-rich layer. Additionally, **Figure S16(c)** demonstrates that the metal-rich layer exhibited a shift toward higher energy loss in the Se low-energy loss spectrum.

Section S10: 4D-STEM results of pristine and biased 1T-TiSe_2_ device

**Figure S17** compares the strain distribution and elemental analysis of pristine and biased 1T-TiSe_2_ devices using 4D-STEM and EDS. **Figure S17(a)-(b)** display HAADF-STEM images of the pristine and biased devices, respectively, showing structural differences. The corresponding EDS point analysis in **Figure S17(c)** confirms a uniform elemental distribution within the pristine TiSe_2_ layers, while **Figure S17(d)** reveals a layered structure and elemental redistribution in the biased device. **Figure S17(e)-(f)** illustrate that the Exx strain remains relatively unchanged before and after biasing. However, the Eyy strain distribution shown in **Figure S17(g)**-(**h)** reveals significant changes, with the biased device exhibiting intensified strain in the upper Ti_9_Se_2_ layer and a more relaxed strain distribution in the lower TiSe_2_ layer. These observations highlight the structural and strain evolution induced by biasing.


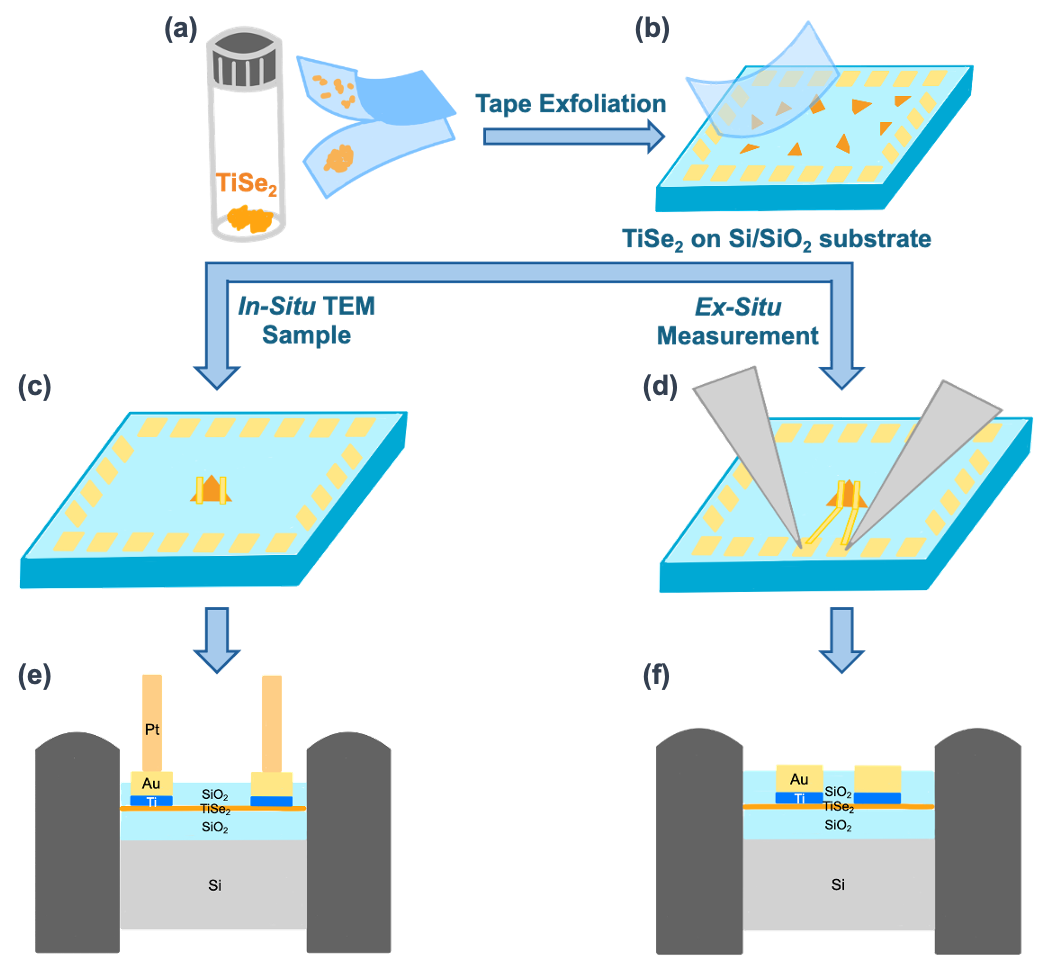


**Figure S1. Experimental setup for *in-situ* and *ex-situ* samples.**

(a)-(b) Bulk TiSe_2_ mechanically exfoliated onto a Si/SiO_2_ substrate, with Ti/Au electrodes at the substrate edges. (c) Fabrication of electrodes for *in-situ* samples using e-beam lithography and an e-gun evaporation system, maintaining an approximate distance of 5 µm between the electrodes. (d) Fabrication of electrodes for *ex-situ* measurement samples using e-beam lithography and an e-gun evaporator, with an approximate distance of 3 µm between the electrodes. (e)-(f) TEM sample preparation using a focused ion beam system.

**
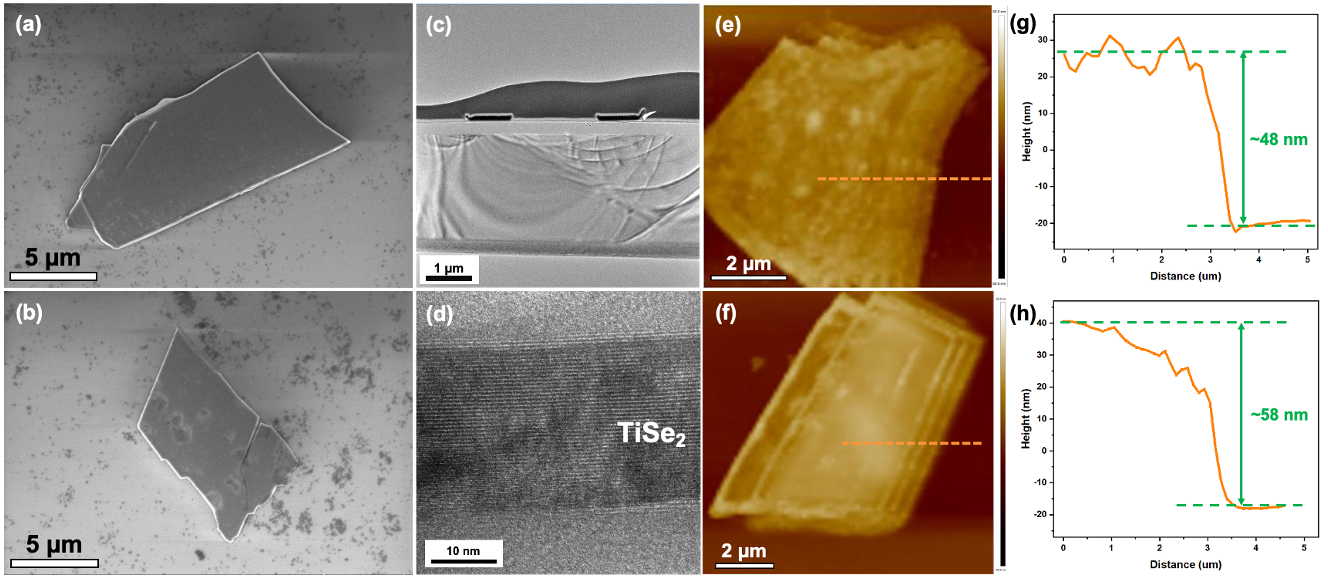
**

**Figure S2. Basic identification of pristine 1T-TiSe_2_.**

(a)-(b) SEM images of a pristine 1T-TiSe_2_ flake. (c) Low-magnification TEM image of the cross-section 1T-TiSe_2_ device. (d) High-magnification TEM image of the cross-section 1T-TiSe_2_ flake. (e)-(f) OM images of a pristine 1T-TiSe_2_ flake. (g)-(h) AFM height profiles of 1T-TiSe₂ flakes along the orange lines in (e) and (f), showing average thicknesses of ≈48 nm and ≈58 nm, respectively.


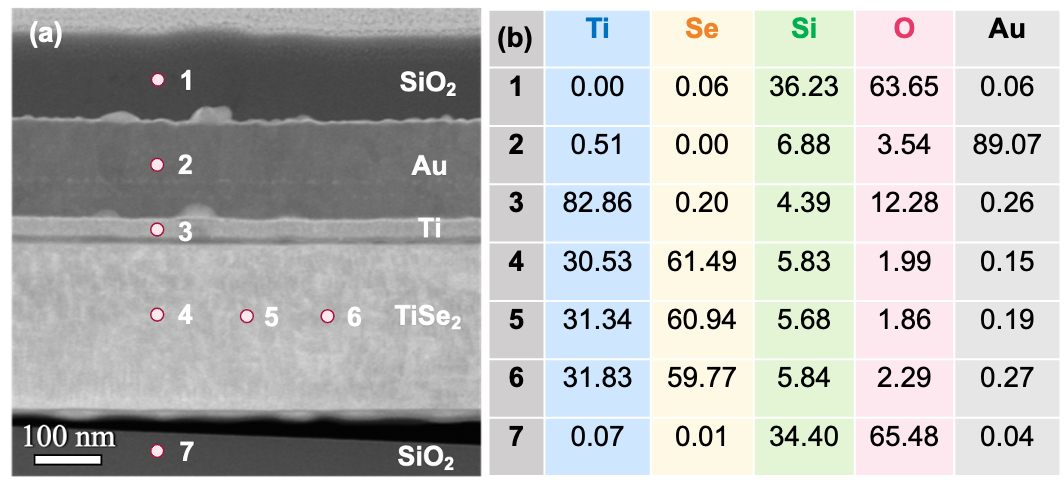


**Figure S3. Characterizations of the pristine cross-section 1T-TiSe_2_ device.**

**(a)** HAADF-STEM image of the pristine cross-sectional 1T-TiSe_2_ device **(b)** EDS point results of a pristine sample, showing the SiO_2_ isolation layer, Ti/Au electrodes, and 1T-TiSe_2_ layer.


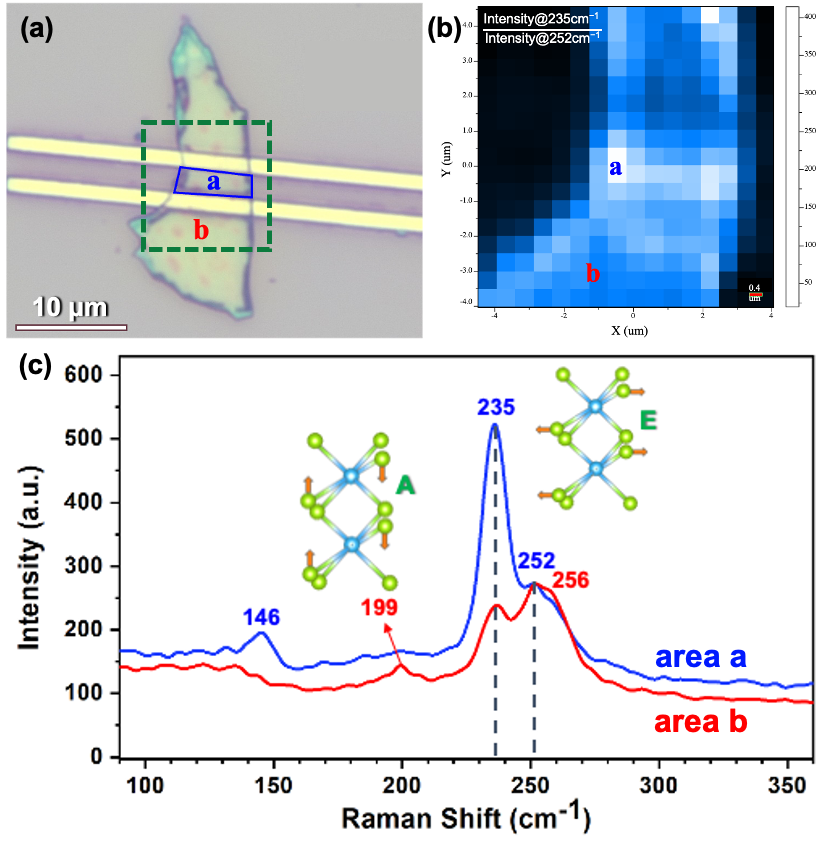


**Figure S4. Raman spectra of the 1T-TiSe_2_ device after *ex-situ* biasing.**

(a) OM image of the 1T-TiSe_2_ device with patterned Ti/Au electrodes; the area between the two electrodes is defined as ‘a’, while the area outside the electrodes is defined as ‘b’. (b) Raman mapping of the biased 1T-TiSe_2_ device in the region highlighted by the green box in (a). (c) Raman spectrum of the 1T-TiSe_2_ device after biasing, showing distinct spectral differences between region 'a' (blue line) and region 'b' (red line).


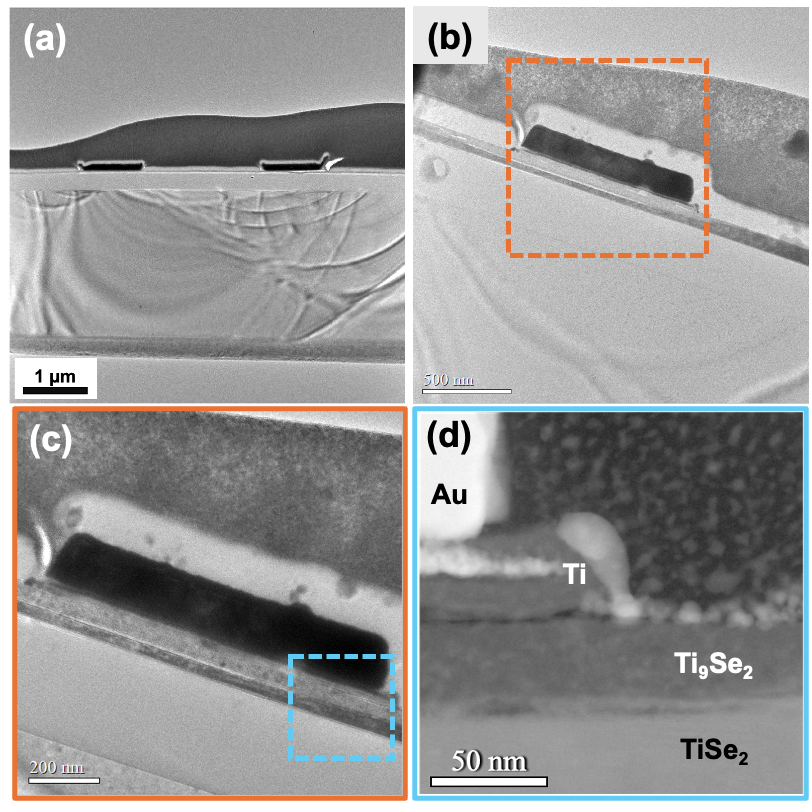


**Figure S5. Detailed TEM images of the cross-sectional 1T-TiSe_2_ device after *ex-situ* electrical measurements.**

(a) Low-magnification TEM image of the *ex-situ* cross-sectional 1T-TiSe_2_ device. (b) Low-magnification TEM image of an electrode in the 1T-TiSe_2_ device. (c) High-magnification TEM image from the orange-boxed region in (b), highlighting the delamination phenomenon of 1T-TiSe_2_ near the electrode after electrical measurements. (d) High-magnification STEM image from the blue-boxed region in (c) , corresponding to the EDS focus area in Figure 2(c).


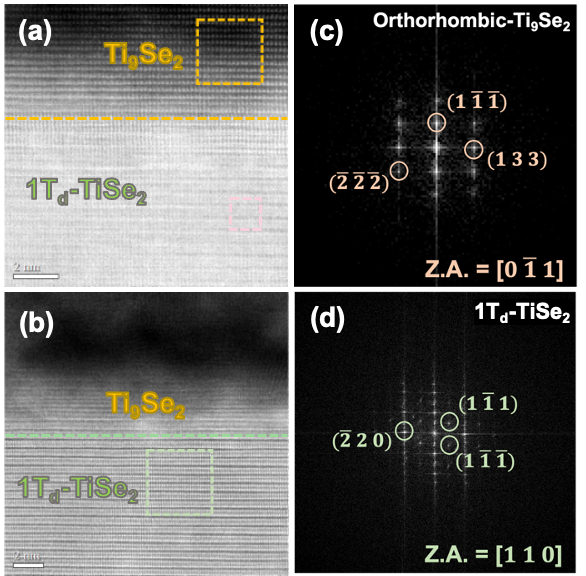


**Figure S6. ADF-STEM images of the *ex-situ* biased 1T-TiSe_2_ device.**

(a)-(b) HAADF-STEM image of a cycled 1T-TiSe_2_ layer, showing regions with differing contrast. (c) Corresponding FFT-DP obtained from the yellow-boxed region in (a), showing the orthorhombic phase Ti_9_Se_2_ along the [$0\bar{1}1$] zone axis. (d) Corresponding FFT-DP obtained from the green-boxed region in (b), showing a distorted structure (1T_d_) along the [110] zone axis.


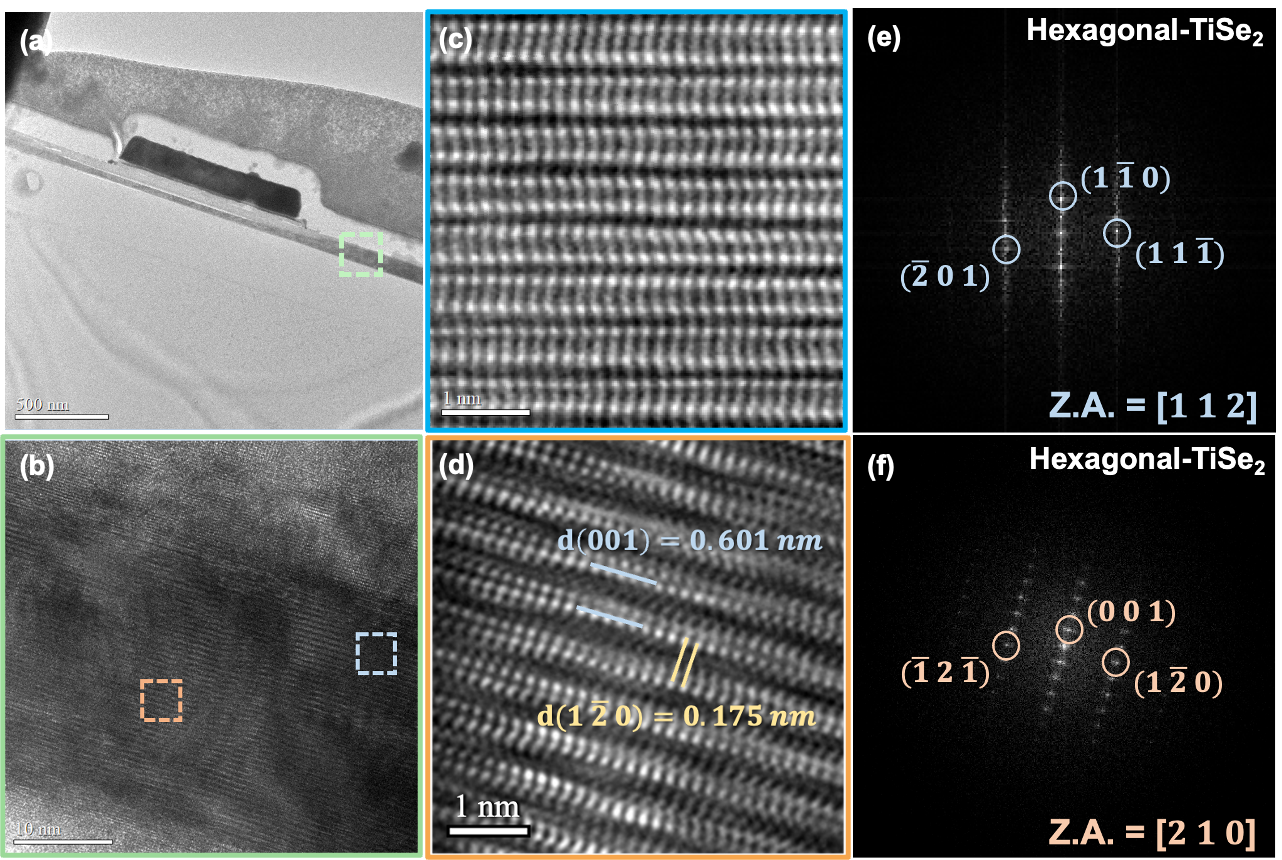


**Figure S7. *Ex-situ* observations of the cross-sectional 1T-TiSe_2_ device away from the electrode.**

(a) Low-magnification TEM image of the cycled sample, showing two contrast regions next to the electrode. (b) High-magnification TEM image from the green-boxed region in (a). (c) HAADF-STEM image from the blue-boxed region in (b), showing no phase changes distant from the Ti/Au electrode. (d) HRTEM image from the orange-boxed region in (b). (e) Corresponding FFT-DP obtained from c, showing the hexagonal phase 1T-TiSe_2_ along the [112] zone axis. (f) Corresponding FFT-DP obtained from (d), showing the hexagonal phase 1T-TiSe_2_ along the [210] zone axis.


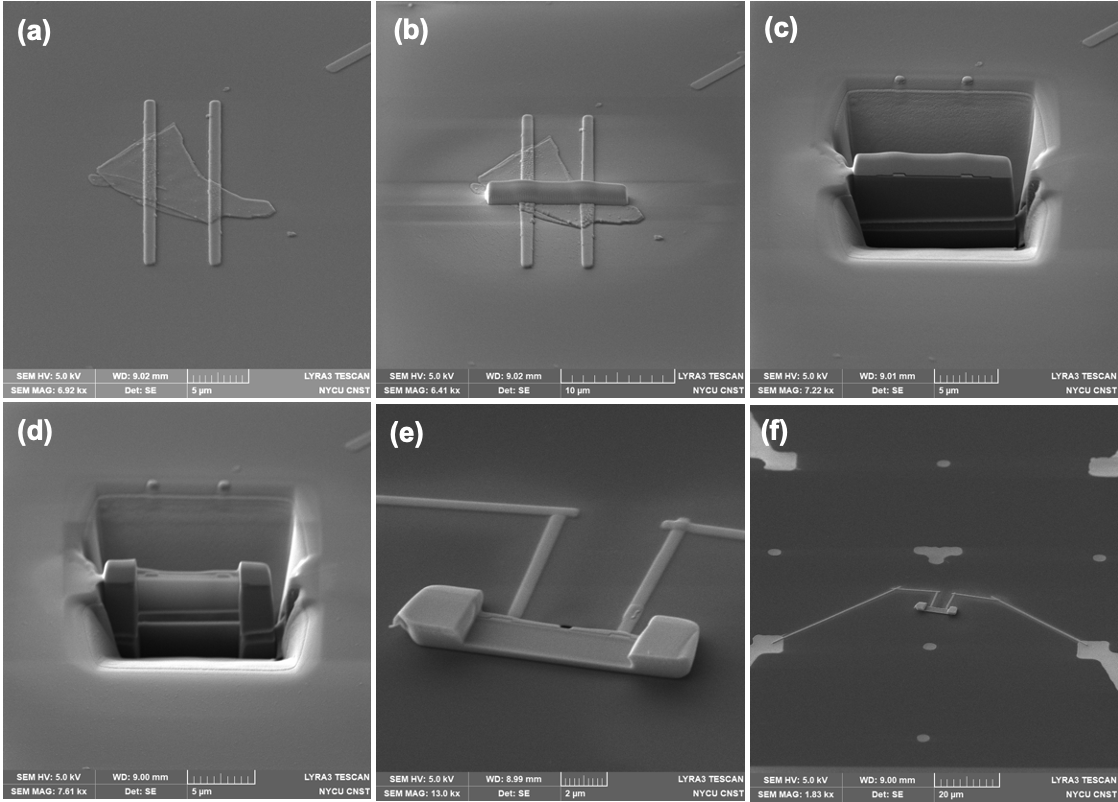


**Figure S8. *Ex-situ* and *in-situ* TEM lamella preparation using the focused ion beam system.**

(a) SEM image of the top view of a 1T-TiSe_2_ flake. (b) Pt protective layer deposited on the device. (c) FIB trench milling process for TEM sample preparation. (d) FIB process for *ex-situ* TEM lamella preparation. (e) Transfer of the *in-situ* TEM sample onto the Si_3_N_4_ membrane using a glass tip. (f) SEM image showing Pt wires deposited to connect the electrodes on the *in-situ* TEM specimen.


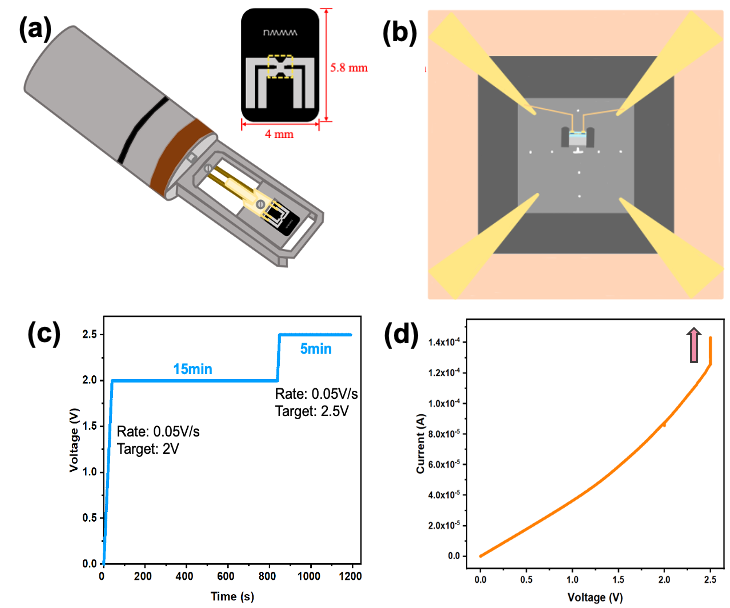


**Figure S9. Experimental setup before biasing and electrical performance of the *in-situ* sample.**

(a) Schematic of the specialized electrifying *in-situ* TEM chip and holder. (b) Enlarged schematic of the electrical chip observation window, with the region indicated using yellow dotted lines in (a). (c) Applied voltage versus time profile of the 1T-TiSe_2_ device. (d) *I–V*curves of CDW-based 1T-TiSe_2_ devices during the *in-situ* biasing experiment.


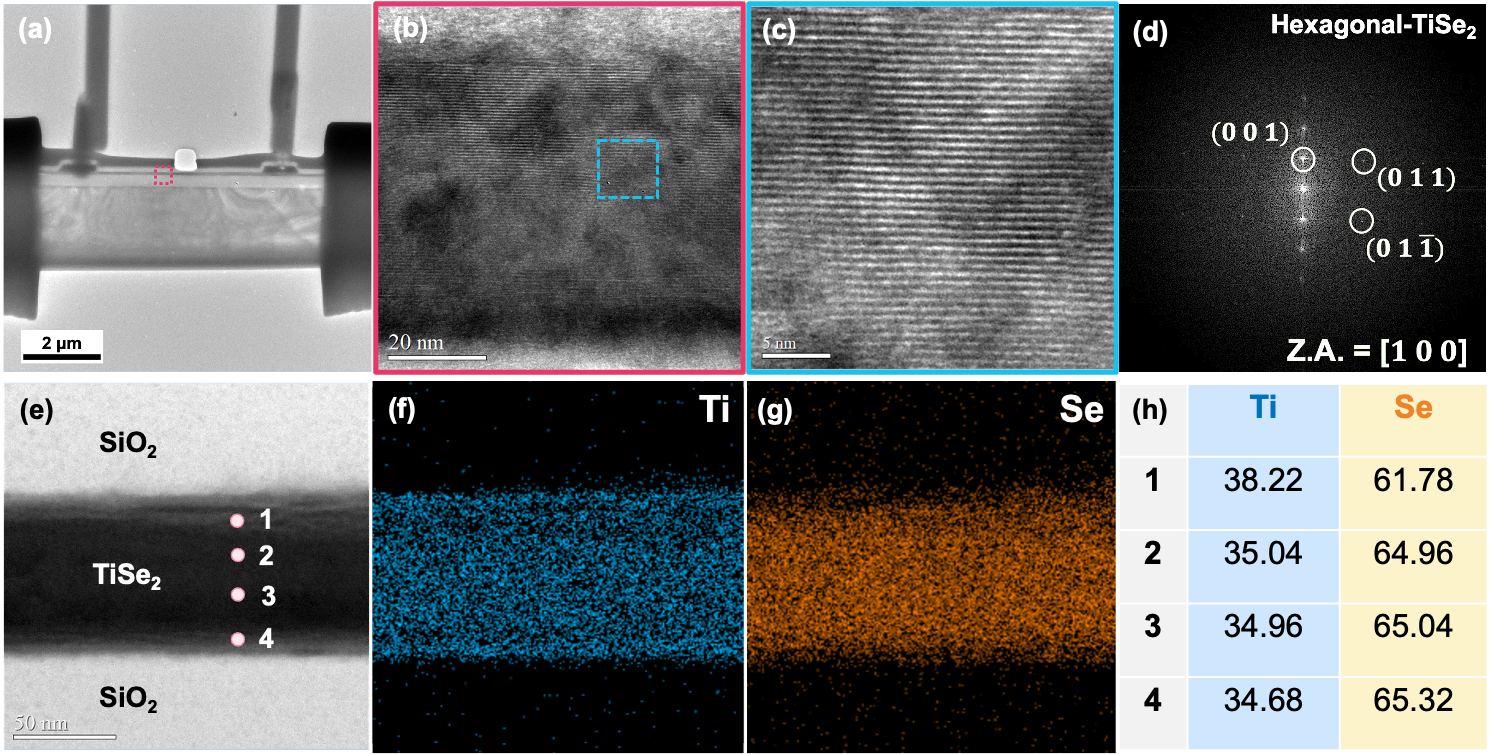


**Figure S10. Initial state of the *in-situ* cross-section 1T-TiSe_2_ device.**

(a) Low-magnification TEM image of the pristine *in-situ* sample. (b) High-magnification TEM image from the pink-boxed region in (a). (c) HRTEM image from the blue-boxed region in (b). (d) Corresponding FFT-DP obtained from c, showing the hexagonal phase 1T-TiSe_2_ along the [100] zone axis. (e) HAADF-STEM image of the pristine 1T-TiSe_2_ device. (f)–(g) Corresponding EDS mapping of the pristine 1T-TiSe_2_ devices, showing the elemental distribution of (f) Ti and (g) Se. (h) EDS point results of the pristine sample in (e).


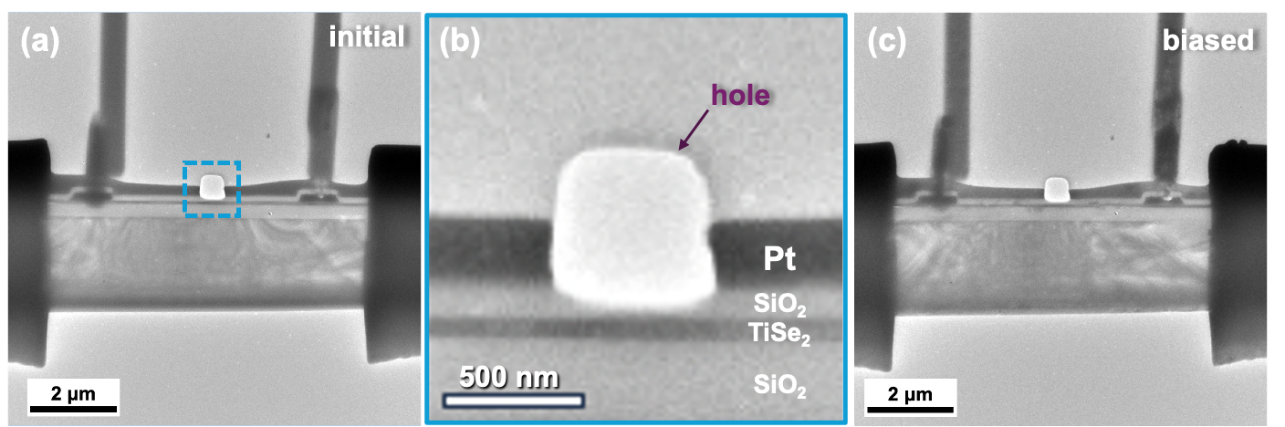


**Figure S11. Schematic illustration of the *in-situ* cross-sectional 1T-TiSe_2_ device.**

(a) Low-magnification TEM image of the initial device. (b) High-magnification TEM image from the blue-boxed region in (a), highlighting the hole created by FIB to disconnect the conduction path in the Pt layer. (c) Low-magnification TEM image of the device after biasing.


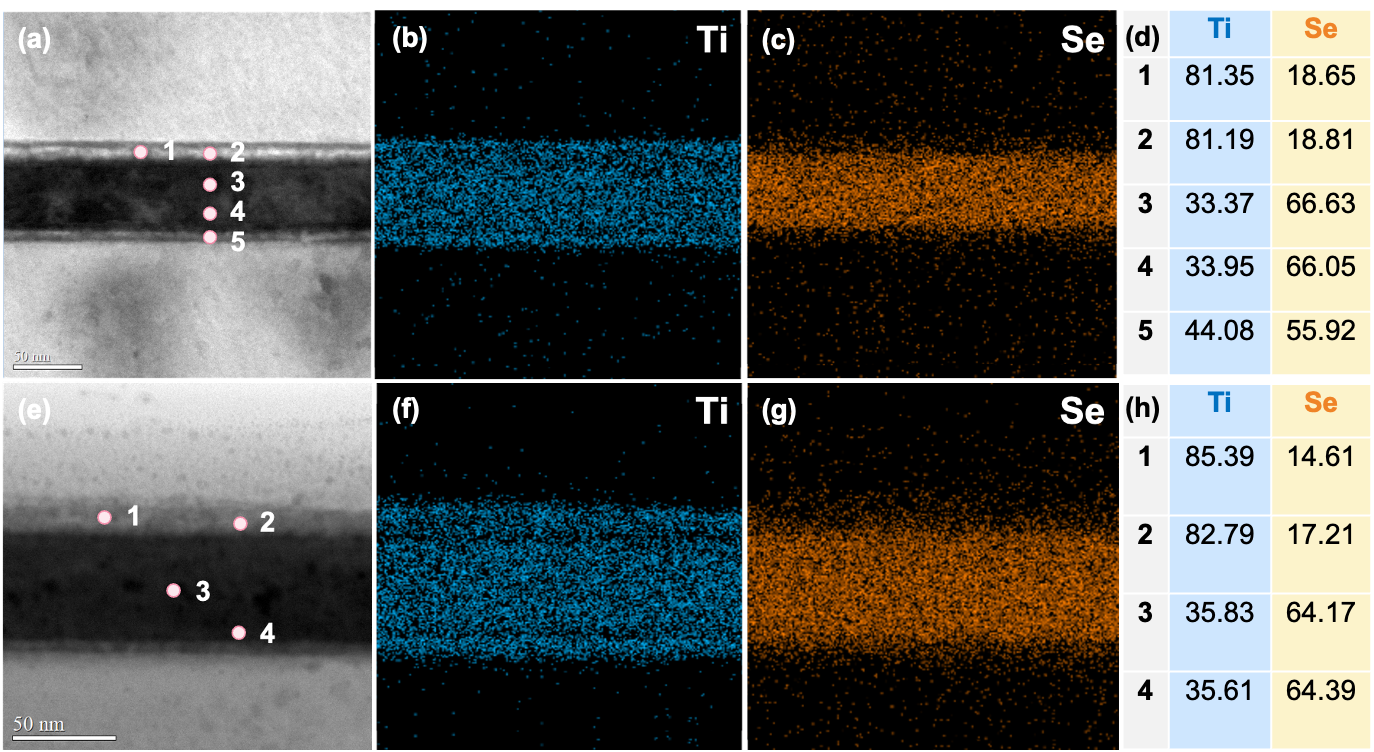


**Figure S12. Elemental quantitative analysis of *in-situ* cross-section 1T-TiSe_2_ device in the non-electron beam irradiated area after biasing.**

(a) Low-magnification HAADF-STEM image of the biased 1T-TiSe_2_ device. (b)-(c) Corresponding EDS mapping in (a), showing the elemental distribution of (b) Ti and (c) Se. (d) EDS point results of the biased sample in (a). (e) High-magnification HAADF-STEM image of the biased 1T-TiSe_2_ device. (f)-(g) Corresponding EDS mapping in (e). (h) EDS point results of the biased sample in (e).


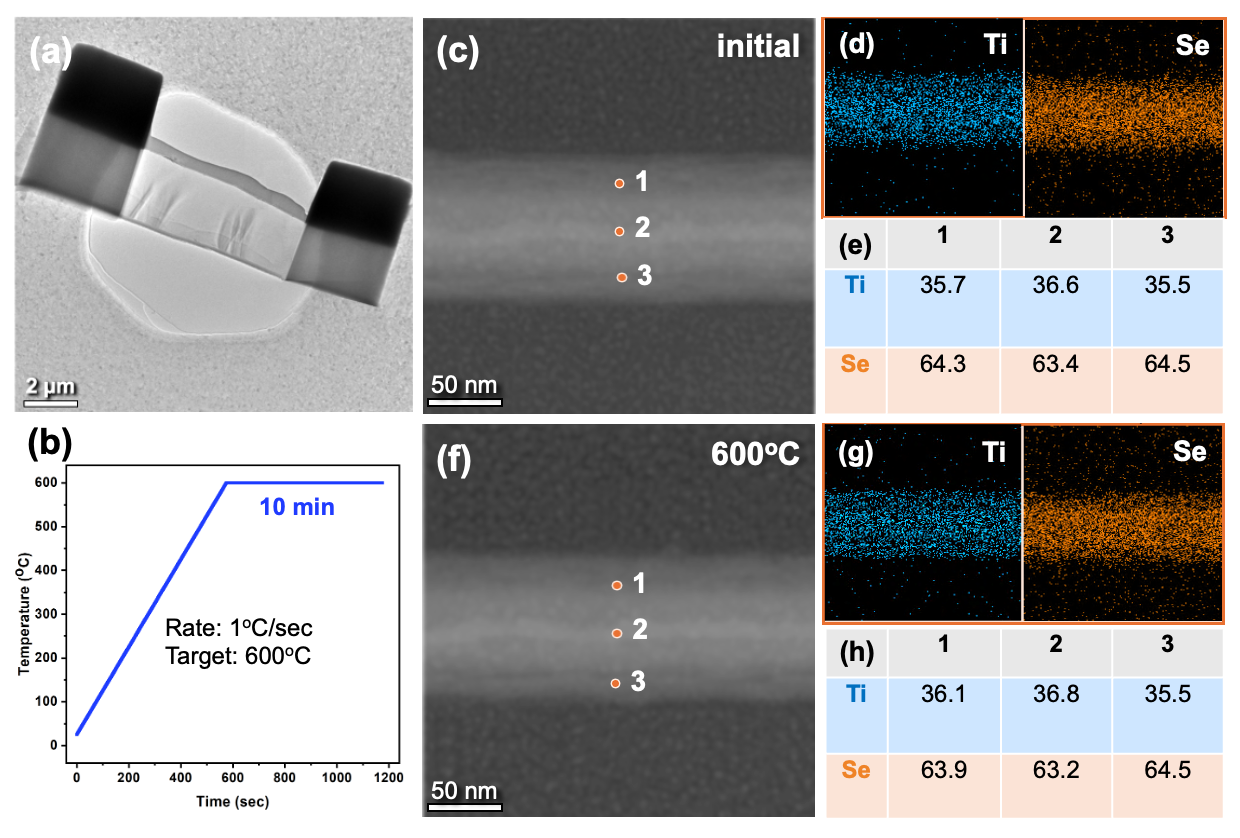


**Figure S13. *In-situ* heating observation of the 1T-TiSe_2_ device.**

(a) Low-magnification TEM image of the *in-situ* cross-sectional 1T-TiSe_2_ device positioned over the hole of the heating chip. (b) Temperature-time profile of the 1T-TiSe_2_ device. (c) High-magnification STEM image of the initial sample. (d) Corresponding EDS mapping of the pristine 1T-TiSe_2_ devices, showing the elemental distribution of Ti and Se. (e) EDS point results of the initial sample in (c). (f) High-magnification STEM image of the sample after *in-situ* heating at 600°C for 10 min. (g) Corresponding EDS mapping, showing the elemental distribution of Ti and Se. (h) EDS point analysis of the sample in (f) after *in-situ* heating at 600°C for 10 min.


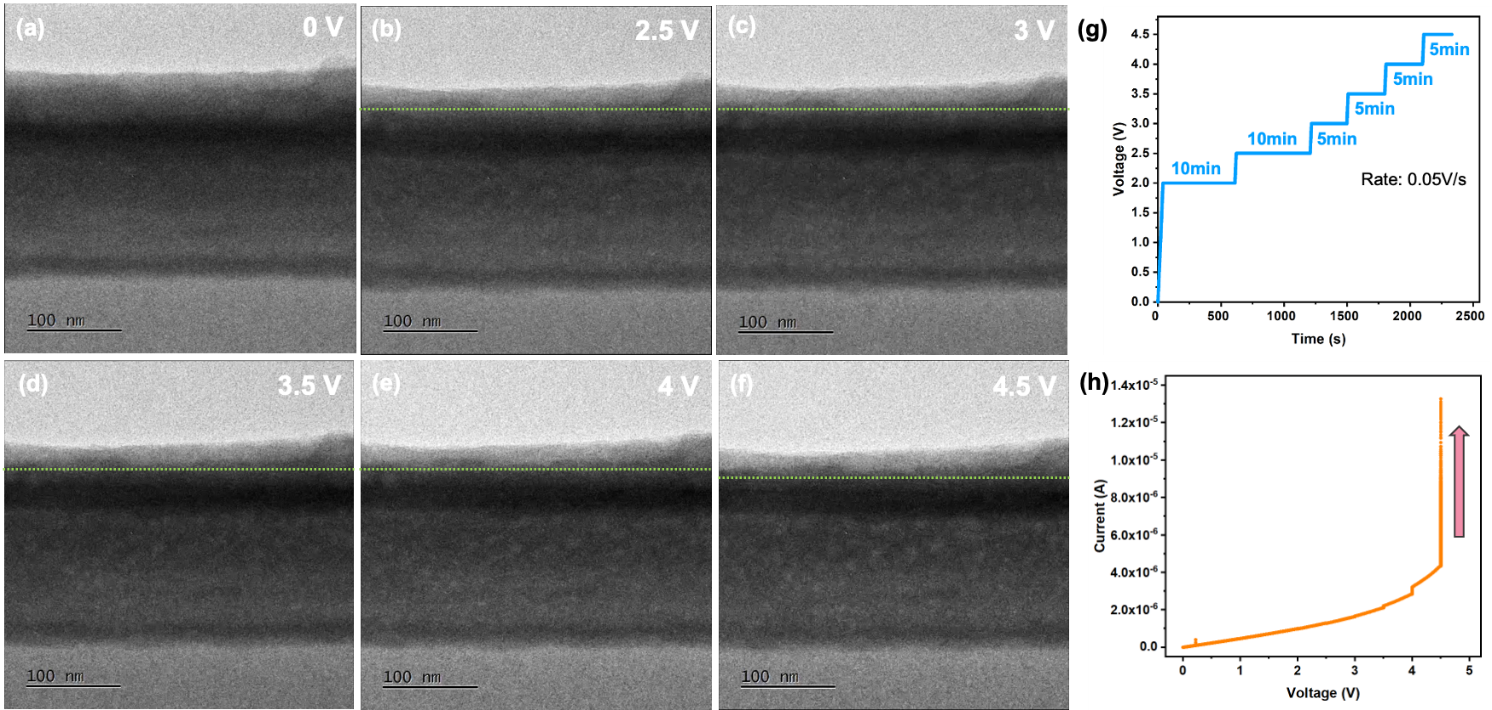


**Figure S14. *In-situ* biasing observation of the 200 nm 1T-TiSe_2_ device.**

(a)-(f) Time-sequenced TEM images showing the phase evolution of the 1T-TiSe_2_ device under a 4.5 V bias. The contrast changes in the 1T-TiSe_2_ layer are highlighted in the green dashed area. (g) Voltage profile over time for the 200 nm 1T-TiSe_2_ device. (h) *I–V* curves of the 200 nm 1T-TiSe_2_ devices recorded during the *in-situ* biasing experiment.


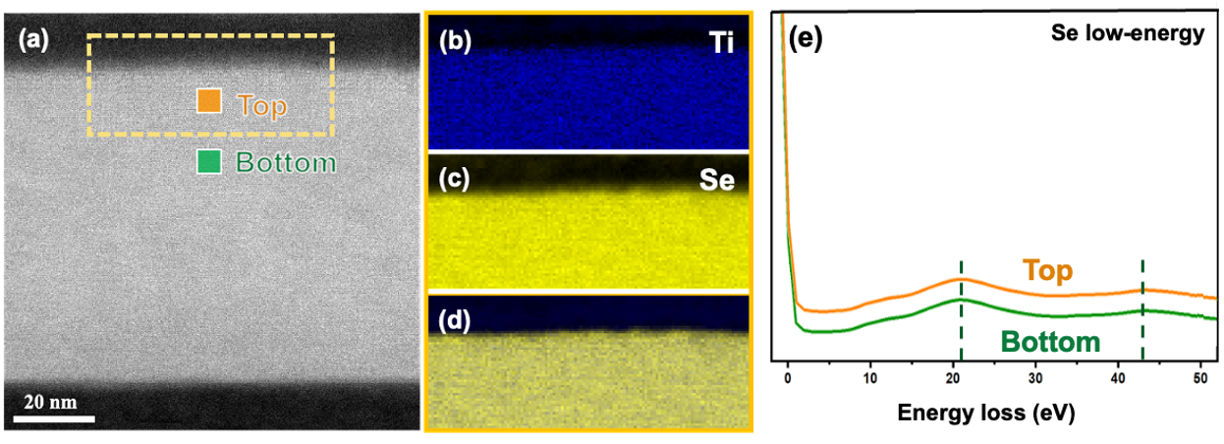


**Figure S15. EELS characterization of the pristine state 1T-TiSe_2_ device.**

(a) STEM image of the pristine state 1T-TiSe_2_ device. Orange and green frames indicate the EELS analysis areas. (b)–(d) EELS mapping from the yellow-boxed region in (a), showing the elemental distribution of (b) Ti, (c) Se, and (d) the overlapping images. (e) EELS spectra of Se low-energy edges in the pristine state 1T-TiSe_2_ device.


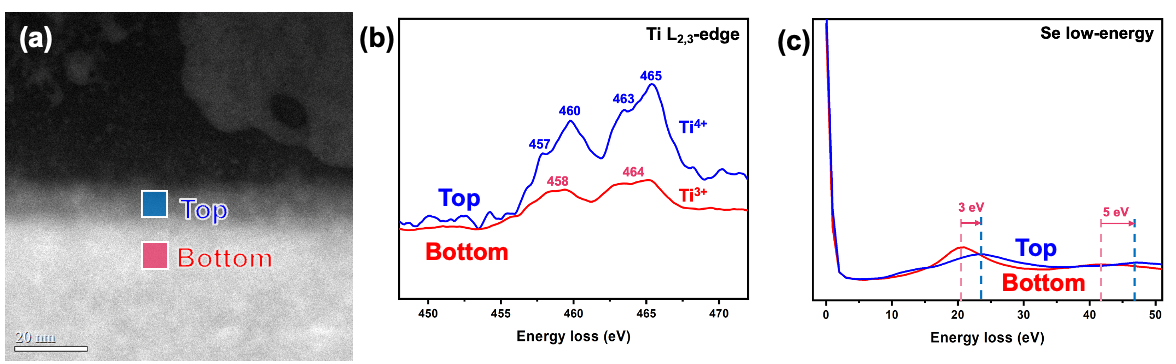


**Figure S16. EELS analysis of the *in-situ* biased 1T-TiSe_2_ device.**

(a) STEM image of the biased 1T-TiSe_2_ device, with blue and red frames indicating the EELS analysis areas. (b) EELS spectra of the Ti-L_2,3_ edges. (c) EELS spectra of Se low-energy edges.


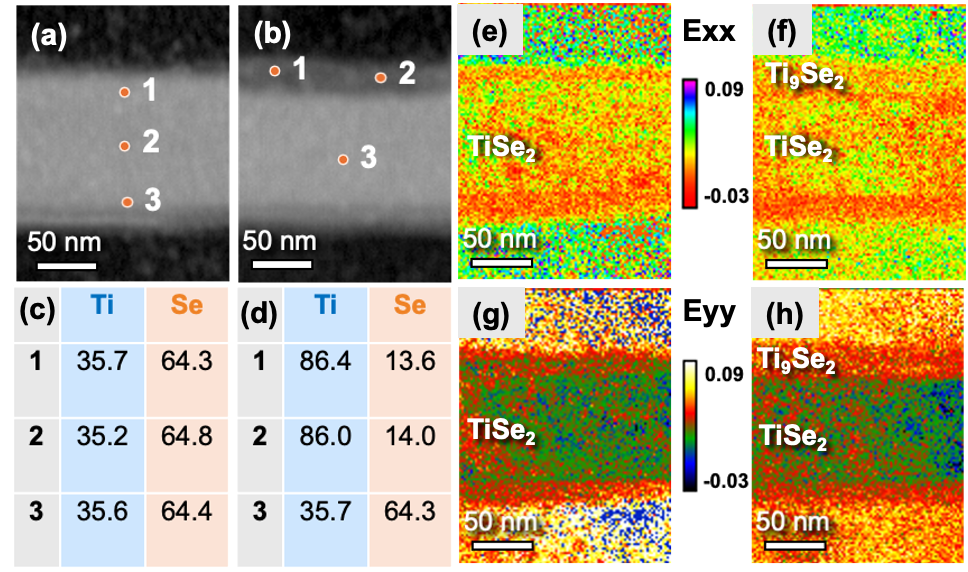


**Figure S17. Comparison of strain in pristine and biased 1T-TiSe_2_ devices using 4D-STEM.**

(a) HAADF-STEM image of the pristine 1T-TiSe_2_ device. (b) HAADF-STEM image of the biased 1T-TiSe_2_ device. (c) The corresponding EDS point analysis in (a), showing the uniform elemental distribution within the original 1T-TiSe_2_ layers. (d) The corresponding EDS point analysis in (b), showing the elemental distribution of the layered structure in the biased 1T-TiSe_2_ device. (e) Strain distribution in the Exx direction of the pristine device at the area shown in (a). (f) Strain distribution in the Exx direction of the biased device at the area shown in (b). (g) Strain distribution in the Eyy direction of the pristine device at the area shown in (a). (h) Strain distribution in the Eyy direction of the biased device at the area shown in (b).
